# Sensory over-responsivity is related to GABAergic inhibition in thalamocortical circuits

**DOI:** 10.1038/s41398-020-01154-0

**Published:** 2021-01-12

**Authors:** Emily T. Wood, Kaitlin K. Cummings, Jiwon Jung, Genevieve Patterson, Nana Okada, Jia Guo, Joseph O’Neill, Mirella Dapretto, Susan Y. Bookheimer, Shulamite A. Green

**Affiliations:** 1 Department of Psychiatry and Biobehavioral Sciences, University of California, Los Angeles, Los Angeles, CA USA; 2 Division of Child & Adolescent Psychiatry, UCLA Jane & Terry Semel Institute for Neuroscience, Los Angeles, CA USA; 3 Department of Psychiatry & The Zuckerman Institute, Columbia University, New York, NY USA

**Keywords:** Pathogenesis, Neuroscience

## Abstract

Sensory over-responsivity (SOR), extreme sensitivity to or avoidance of sensory stimuli (e.g., scratchy fabrics, loud sounds), is a highly prevalent and impairing feature of neurodevelopmental disorders such as autism spectrum disorders (ASD), anxiety, and ADHD. Previous studies have found overactive brain responses and reduced modulation of thalamocortical connectivity in response to mildly aversive sensory stimulation in ASD. These findings suggest altered thalamic sensory gating which could be associated with an excitatory/inhibitory neurochemical imbalance, but such thalamic neurochemistry has never been examined in relation to SOR. Here we utilized magnetic resonance spectroscopy and resting-state functional magnetic resonance imaging to examine the relationship between thalamic and somatosensory cortex inhibitory (gamma-aminobutyric acid, GABA) and excitatory (glutamate) neurochemicals with the intrinsic functional connectivity of those regions in 35 ASD and 35 typically developing pediatric subjects. Although there were no diagnostic group differences in neurochemical concentrations in either region, within the ASD group, SOR severity correlated negatively with thalamic GABA (*r* = −0.48, *p* < 0.05) and positively with somatosensory glutamate (*r* = 0.68, *p* *<* 0.01). Further, in the ASD group, thalamic GABA concentration predicted altered connectivity with regions previously implicated in SOR. These variations in GABA and associated network connectivity in the ASD group highlight the potential role of GABA as a mechanism underlying individual differences in SOR, a major source of phenotypic heterogeneity in ASD. In ASD, abnormalities of the thalamic neurochemical balance could interfere with the thalamic role in integrating, relaying, and inhibiting attention to sensory information. These results have implications for future research and GABA-modulating pharmacologic interventions.

## Introduction

Sensory processing of environmental stimuli is a core function of the central nervous system and an important determinant of behavioral output. Successful navigation and interaction with the environment require appropriate interpretation and regulation of responses to sensory information. Sensory over-responsivity (SOR)—responding too much, for too long, or to stimuli of weak intensity—represents a major disruption in these processes and is exhibited by sensitivity to or avoidance of sensations such as loud or unpredictable noises, visually stimulating environments, or scratchy clothing tags^1^. SOR is present in 5–15% of the general population^2^ and is even more common (rates over 50%) in individuals with both genetic and environmentally-based psychiatric and neurodevelopmental disorders such as anxiety, attention deficit hyperactivity disorder, early life adversity, and autism spectrum disorder (ASD)^3–5^. Across ages and diagnostic groups, SOR is associated with decreased participation in daily life activities, greater psychological distress, and family impairment^5–7^. SOR has been most studied in ASD and atypical sensory processing was recently included as one of the core features of ASD in the DSM-5 (Diagnostic and Statistical Manual of Mental Disorders, 5^th^ edition). While up to 70% of children and adolescents with ASD meet criteria for SOR^8,9^, it is an important source of phenotypic variability in this population and is associated with the severity of functional impairment, deficits in social and adaptive skills, and anxiety^10,11^.

The thalamus is traditionally considered a subcortical relay for sensory information from periphery to cortex. More recent work has demonstrated the important role of the thalamus in filtering and organizing sensory information and of thalamocortical circuits implicated in attentional control of sensory information by modulating and sustaining functional interactions within and between cortical areas^12^. Thalamic inhibition is a critical element of thalamocortical filtering and attentional control that comprises circuits acting across spatial and temporal domains. This inhibition is established through GABAergic (gamma-aminobutyric acid) regulation of thalamocortical circuits.

While the neural mechanisms underlying SOR are not fully understood, the thalamus is expected to play a key role given its prominence in sensory filtering and attention. Existing SOR studies indicate both bottom-up and top-down regulation processes in SOR. Recent fMRI research suggests that in ASD, SOR is associated with over-reactive brain responses to sensory input in primary sensory processing areas of the brain (e.g., auditory and somatosensory cortices), as well as in brain areas related to affective valence, salience, and attention to information (e.g., amygdala, insula, and thalamus)^13,14^. Furthermore, youth with ASD and SOR show decreased neural habituation to sensory stimuli and lack prefrontal inhibition of the amygdala during exposure to mildly aversive sensory stimuli^13^. Likewise, they show reduced thalamocortical modulation in response to sensory stimulation compared to typically developing (TD) youth. Specifically, TD youth showed greater functional connectivity of the pulvinar with sensory-motor and prefrontal regions, whereas ASD participants, particularly those with higher SOR, demonstrated greater pulvinar-amygdala connectivity^15^. Taken together, these findings suggest that SOR is related to bottom-up, primary sensory processing abnormalities along with top-down deficits in thalamocortical inhibition, which could lead to difficulty habituating to, filtering out, and/or integrating sensory information. In turn, individuals with SOR would then attribute more salience and attention to extraneous sensory stimuli, at the cost of normative social orienting^16^.

The mechanisms underlying these alterations in thalamocortical circuits in ASD are poorly understood but may be related to abnormalities in thalamic excitatory-inhibitory (E/I) neurochemical balance^17^, which could interfere with the thalamic role in integrating, relaying, and inhibiting attention to sensory information. Cortical and thalamic E/I imbalances have been implicated in the pathogenesis of a wide range of neurodevelopmental and neuropsychiatric disorders including epilepsy, schizophrenia, depression, Alzheimer’s disease, and ASD^18–20^. Across and within these disorders, alterations in E/I ratio are associated with distinct and shared genetic etiologies and behavioral phenotypes pointing to the importance of understanding cross-diagnostic mechanisms in E/I balance^21^. Additionally, the timing of E/I imbalance in relation to neurodevelopment is likely a major factor in phenotypic variation^20^. Theoretical and computational models of autism suggest that an increased excitatory-inhibitory ratio may cause cortical hyper-reactivity and/or changes in endogenous neural noise that could negatively impact developmental processes important to sensory system tuning, such as lateral inhibition^22–24^.

GABA and glutamate are the primary inhibitory and excitatory neurotransmitters of the CNS, respectively. Currently, the only non-invasive method to measure in vivo glutamate and GABA levels is proton magnetic resonance spectroscopy (^1^H-MRS)^25^. Thereby, glutamate is frequently assayed as “Glx”, the sum of glutamate and glutamine, and GABA as “GABA+”, the sum of GABA and spectrally overlapping macromolecules. ^1^H-MRS studies comparing children, adolescents, and adults with ASD to neurotypical peers have yielded diverse findings in Glx and GABA+ in various cortical and subcortical regions^26–28^. While GABA+ clearly trends towards below-normal or normal levels in ASD children or adults^26–34^, Glx findings are inconsistent in ASD and neurotypical samples^26–31,35–37^. Several studies have found that somatosensory cortex GABA+ levels correlate with greater precision in tactile detection threshold tasks in children^34^ and adults^32,38^. Sapey-Triomphe et al. further reported a positive correlation between self-reported tactile hypersensitivity and somatosensory GABA in ASD but not neurotypical adults and suggested that altered GABA modulation in ASD is associated with decreased predictability of tactile stimuli and, therefore, hyper-reactivity^32^.

Just as there is wide variability in findings of neurotransmitter concentrations in ASD studies, among individuals with ASD who experience SOR, there is vast heterogeneity in severity of reported discomfort and ability to regulate response to unpleasant sensory stimuli. Given our previous finding of abnormally modulated thalamocortical and thalamo-amygdala connectivity in response to sensory stimuli in ASD, we hypothesized that E/I neurochemical balance could underlie SOR heterogeneity in ASD such that top-down sensory modulation by thalamic inhibition would be related to both behavioral measures of SOR and intrinsic functional brain connectivity with regions known to be over-reactive in subjects with SOR. We further expected that the E/I relationship of the thalamus with SOR would differ from that observed in primary sensory cortex as these areas differ in the extent to which they exert top-down attention and filtering control versus bottom-up local processing alterations in modulating sensory responses.

## Materials and Methods

### Participants

Participants were children and adolescents aged 8–17 years—35 with ASD (confirmed with Autism Diagnostic Interview–Revised^39^, Autism Diagnostic Observation Schedule^40^, and clinical judgment) and 35 typically-developing (TD; Table 1). Groups did not differ significantly for sex (*χ*^*2*^ *ns*) or head motion during fMRI (eTable 1). The ASD group was significantly older than the TD group (*p* < 0.05). All participants had a full-scale IQ > 85 on the Weschler Abbreviated Scales of Intelligence (WASI)^41^ with no significant group difference on performance IQ but significantly lower verbal and full-scale IQ (FSIQ) in the ASD sample (Table 1). Therefore, age and FSIQ were used as covariates in all group comparisons and in within-group regressions where *p* < 0.10. Additional group comparisons using an age-matched TD subgroup yielded similar results to those presented here (eTable 2). Among the ASD youth, 15 were taking psychotropic medications (SSRI *n* = 8, stimulant *n* = 8, **α**_**2**_ agonist *n* = 4, antipsychotic *n* = 4, antihistamine *n* = 3). No TD youth were taking psychotropic medications (eTable 3).

**Table 1:** Demographics and behavioral measures.

	ASD (mean ± SD)	TD (mean ± SD)	*t* or χ^2^
Subjects (number)	35	33	
Sex (females)	9	12	*p* = 0.34
Age (years)	14.57 ± 2.7	13.09 ± 3.0	*p* = 0.036
Social responsivity scale (SRS) Total	76.11 ± 27.4	19.42 ± 17.5	*p* < 0.001
Anxiety total score (SCARED Parent)	18.57 ± 11.7	6.03 ± 6.5	*p* < 0.001
WASI full-scale IQ	106.57 ± 15.2	116.82 ± 12.6	*p* < 0.01
WASI verbal IQ	102.51 ± 17.4	116.97 ± 13.2	*p* < 0.001
WASI performance IQ	109.97 ± 15.9	112.70 ± 12.8	*p* = 0.38
SOR total score (SenSOR parent)	75.49 ± 25.9	49.09 ± 7.0	*p* < 0.001

ASD Autism spectrum disorder, SCARED Screen for child anxiety related emotional disorders, SenSOR Sensory over-responsivity inventory, TD Typically developing, WASI Weschler Abbreviated Scales of Intelligence.

Procedures were approved by the University of California, Los Angeles, Institutional Review Board. Written informed consent was obtained from all parents and participants 13 years or older. Written assent was obtained from participants younger than 13 years. The study was conducted between November 2017 and November 2019.

### Behavioral measures

Diagnostic and cognitive measures were administered at a clinical assessment visit. Child anxiety (Screen for Child Anxiety Related Emotional Disorders; SCARED) and sensory responsivity (Sensory Over-Responsivity Inventory; SensOR Inventory) questionnaires were completed by parents (Table 1). The SCARED is a 41-item parent report questionnaire of child anxiety symptoms^42^. The total score was used as a continuous measure of anxiety symptom severity. The SCARED has been validated in ASD populations and has been shown to have good internal consistency, test-retest reliability, and discriminative validity^43,44^. Here, as shown elsewhere^10,45^, anxiety correlated significantly with SOR (all subjects *r* = 0.63, *p* < 0.001; ASD *r* = 0.51, *p* < 0.01) and hence was covaried in all SOR analyses to identify unique effects beyond anxiety.

The SensOR Inventory is a caregiver rating scale of sensory sensations that bother their child^46^. For this study, the auditory, visual, and tactile subscales were used. The number of items parents rate as bothering their child has been shown to discriminate between children with and without SOR. Here, the checklist items were scored with a 5-point Likert scale ranging from 0 for “not bothersome or does not avoid” to 4 for “very bothersome or always avoids”. Scores for all questions related to sensory over-responsivity were summed to yield a total score.

### MR Acquisition

Magnetic resonance scans were acquired on a 3-Tesla MRI system (Siemens Prisma, Erlangen, Germany) equipped with a 64-channel head coil. A high-resolution T1-weighted image was collected for positioning the ^1^H-MRS VOIs (MPRAGE sequence, FOV 250 mm × 250 mm; slice thickness 0.85 mm; matrix: 288 × 288; 208 slices; TR 1 800 ms; TE 2.37 ms; TI 900 ms; flip angle 8°; parallel imaging (GRAPPA 3); scan time of 3 min 39 s). Single-voxel edited ^1^H-MR spectra were collected from two brain regions (“volumes-of-interest, VOIs”) using a Siemens prototype MEGA‐PRESS sequence (TE/TR 68/2 000 ms; bandwidth 1 200 Hz; 512 data points; 128 × 2 averages), with selective inversion pulses applied at 1.9 ppm (On) and at 7.5 ppm (Off)^47^. The VOIs were in: (1) the bilateral thalamus (volume = FH 25 mm × LR 35 mm × AP 10 mm = 8 750 mm^2^; see eFig. 1.A); and, (2) the right post-central gyrus with hand knob positioned centrally (volume = 20 mm × 30 mm × 20 mm = 12,000 mm^2^; see eFig. 1.B).

Functional magnetic resonance imaging (fMRI) resting-state scans were acquired while participants fixed their gaze on a white crosshair on a black background, presented using a pair of 800 × 640 resolution magnet-compatible 3-D goggles under computer control (Resonance Technology Inc., Northridge, CA, USA). Scans were acquired using an EPI multi-band acquisition lasting 8 minutes and covering the entire cerebral volume (TR = 720 ms, FOV = 208 mm, TE = 37 ms, flip angle = 52°, in-plane voxel size = 2 mm^2^, 72 slices, multi-band acceleration factor = 8).

### ^1^H-MRS Analysis

Edited ^1^H-MR spectra were acquired as above and analyzed utilizing the Gannet pipeline^48^ with difference editing per Guo et al. ^49^ yielded GABA+/Cr and Glx/Cr as endpoints. ^1^H-MRS post-processing was conducted in MATLAB and consisted of spectral phase and frequency correction, difference editing^49,50^, and frequency-domain peak fitting with a simulated basis-set^51^ using the modeling algorithms proposed by the Gannet pipeline^48^ yielding high accuracy GABA+ macromolecules (GABA+) and glutamate+glutamine (Glx) signal intensities. For both VOIs, off-pulse creatine linewidths were less than 10 Hz and difference spectra fit errors greater than 20% were not used. This resulted in: 29 ASD and 31 TD subjects with useable thalamus ^1^H-MR spectra; 21 ASD and 23 TD subjects with useable SSC ^1^H-MR spectra; and, 14 ASD and 18 TD subjects with data from both regions.

Using Gannet scripts, the ^1^H-MRS VOIs were co-registered with the tissue-segmented structural image (MPRAGE; SPM8, Wellcome Trust Center for Neuroimaging) to calculate tissue fractions in each VOI. The VOI tissue segmentation fractions of gray matter (GM), white matter (WM), and cerebrospinal fluid (CSF) were not significantly different between groups for either VOI (eTable 1). The tissue fractions of each VOI did not correlate with age or full-scale IQ. The only metabolite that correlated with tissue fraction was SSC Glx/Cr with GM fraction, so GM fraction was used as a covariate for associated regressions.

### fMRI Analysis

The resting state functional MRI (rsfMRI) data were analyzed using the FMRIB Software Library (FSL), version 5.0.10 (www.fmrib.ox.ac.uk/fsl). 2 TD and 1 ASD subjects were excluded due to maximum motion >4 mm and 3 TD subjects did not have rsfMRI data. The preprocessing pipeline included spatial smoothing (Gaussian kernel full width at half maximum = 5 mm), bandpass filtering (0.1 Hz > *t* > 0.01 Hz), and the regression of mean white matter, cerebrospinal fluid, and global signal times series. Independent Component Analysis-Automatic Removal of Motion Artifacts (ICA-AROMA)^52^ was used to remove potential confounds resulting from head motion by regressing out single-subject components labeled as motion or noise. Each participant’s data were then registered to the MNI152 T1 2 mm template brain (12 degrees of freedom).

Two seeds were used for resting state connectivity analyses. The thalamus seed was the Harvard-Oxford probabalistic cortical atlas provided with FSL^53^ bilateral thalamus at 50% threshold (population probability map). The SSC was the Harvard-Oxford Cortical Atlas bilateral post-central gyrus at 50% threshold. A fixed-effects model was run for each individual subject using FSL’s fMRI Expert Analysis Tool (FEAT, version 6.0). For each seed, connectivity maps were created by isolating the time-series from that region and correlating it with activity of every other voxel in the brain in order to find regions of synchronous activity. Fischer’s r-to-z transformation was then used to create z-statistic maps. Individual FEATS were combined in higher-level mixed-effects model group analyses that were conducted using FSL’s Local Analysis of Mixed Effects (FLAME 1 + 2)^54,55^. Within and between-group comparisons were voxelwise thresholded at *Z* > 2.3 (*p* < 0.01) and whole-brain cluster corrected at *p* < 0.05. To examine how thalamus connectivity related to thalamus GABA concentration, GABA+/Cr measures were entered as a bottom-up regressor in the whole-brain analysis. To examine how SSC connectivity related to SSC Glx concentration, Glx/Cr measures were entered as a bottom-up regressor in the whole-brain analysis. There were no significant correlations between cluster functional connectivity measures and resting state scan motion in either group (corrected for multiple comparisons).

### Statistical Analyses

Based on our pilot MRS data (metabolite correlations with SOR ranged from 0.52 to 0.89) and MRS data collected by other groups^26,37,56^, we predicted that groups of 25 ASD and 25 TD youth would provide 83–100% power to detect effects of metabolites relating to SOR in ASD and 80% power to detect an effect size of Cohen’s d = 0.80 for between-group differences in metabolites. Metabolite and behavioral measures were tested for normality using a one-sample Kolmogorov–Smirnov test (KS-test): SOR is normally distributed in the ASD group (KS-test = 0.13, *p* = 0.18); and, both thalamic GABA+/Cr (KS-test = 0.90, *p* = 0.39) and somatosensory cortex Glx/Cr (KS-test = 1.22, *p* = 0.10) are normally distributed.

Regression analyses and group mean differences were performed in SPSS (IBM Corp., Armonk, NY, USA). Metabolite endpoints were tested for correlations with age, fraction cerebrospinal fluid (fCSF), and fraction gray matter (fGM); SOR was tested for correlations with age, SCARED, and IQ. Those measures that predicted metabolites or SOR at *p* < 0.10 were used as covariates in multiple linear regression analyses of metabolites, SOR, and resting-state measures. For all group differences, Levene’s test for equality of variances was performed and *t*-tests were two-sided. Pearson’s *r* coefficients were compared as dependent correlations with overlapping variables using R^57^ and R package cocor^58^.

## Results

### Relationship between neurometabolites and behavioral measures

Independent-sample *t*-tests showed no significant between-group differences in GABA+/Cr, Glx/Cr or GABA+/Glx in either thalamus or somatosensory cortex (SCC) while ASD showed significantly greater variance in SCC Glx/Cr (*p* < 0.05) compared to TD (Table 2). GABA+/Cr and Glx/Cr were not correlated with age, IQ, or anxiety in either group. SOR was not significantly correlated with age or IQ in either group. SOR was significantly correlated with anxiety and anxiety was thus included as a covariate in all SOR analyses.

**Table 2:** ^1^H-MRS Metabolite Data.

	ASD (mean ± SD)	TD (mean ± SD)	*t*-test of means	*F* test of variances
*Subjects with useable Thalamus* ^*1*^*H-MRS*^a^	*29*	*31*		
Thalamus Metabolite Concentrations				
Creatine (i.u. × 10^–6^)	2 ± 0.19	2 ± 0.14	*t* = −1.04, *p* = 0.30	*F* = 1.07, *p* = 0.31
GABA+/Creatine	0.20 ± 0.06	0.21 ± 0.09	*t* = −0.56, *p* = 0.58	*F* = 1.97, *p* = 0.17
Glx/Creatine	0.12 ± 0.05	0.12 ± 0.06	*t* = 0.27, *p* = 0.79	*F* = 0.01, *p* = 0.94
GABA+/Glx	1.86 ± 0.92	1.99 ± 1.23	*t* = −0.46, *p* = 0.65	*F* = 0.01, *p* = 0.94
Subjects with useable Right SS Cortex ^1^H-MRS^b^	*21*	*23*		
Right SS Cortex Metabolite Concentrations				
Creatine (i.u. × 10^–6^)	4 ± 0.63	4 ± 0.52	*t* = −1.68, *p* = 0.10	*F* = 0.27, *p* = 0.60
GABA+/Creatine	0.17 ± 0.07	0.14 ± 0.05	*t* = 1.42, *p* = 0.16	*F* = 1.74, *p* = 0.20
Glx/Creatine	0.15 ± 0.03	0.14 ± 0.02	*t* = 0.36, *p* = 0.78	*F* = 6.95 *p* = 0.012*
GABA+/Glx	1.23 ± 0.06	1.05 ± 0.04	*t* = 1.18, *p* = 0.24	*F* = 1.49, *p* = 0.23

a Demographic and behavioral measures for this sub-group were not substantially different from the full group in Table 1.

b Demographic and behavioral measures for this sub-group were not substantially different from the full group in Table 1 with the exception of age (ASD mean = 13.5, TD mean = 12.7, *ns*).

*p < 0.05, ASD Autism spectrum disorder, ^1^H-MRS Proton magnetic resonance spectroscopy, GABA+ gamma-aminobutyric acid + macromolecules, Glx glutamate + glutamine, i.u. institutional units, SS somatosensory, TD Typically developing.

**Fig. 1:**
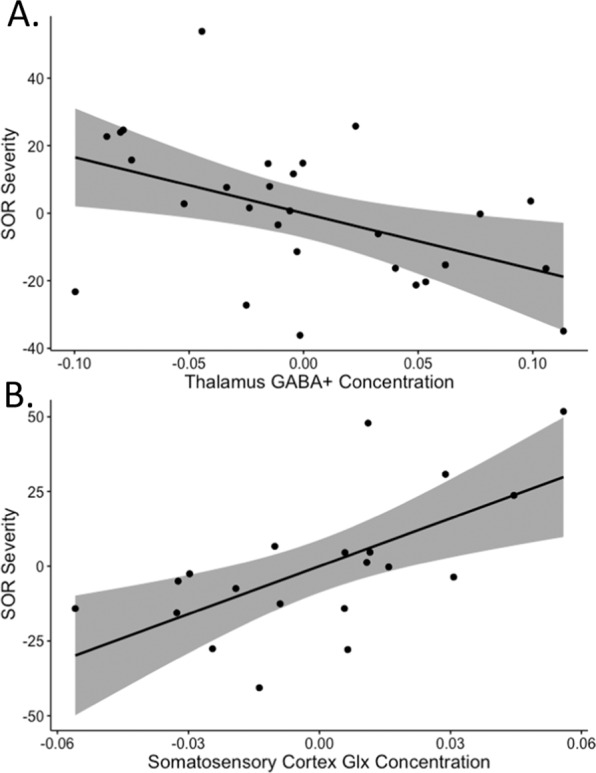
Relationship between Sensory Over-Responsivity Severity and Neurometabolites in ASD Youth. SOR severity scores are significantly correlated with: **A** Thalamus GABA+/Cr (*r* = −0.48, *p* < 0.05, *n* = 28; plot of partial regression corrected for SCARED); and, **B** SSC Glx/Cr (*r* = 0.68, *p* < 0.01, *n* = 20; plot of partial regression corrected for SSC voxel tissue fraction and SCARED).

SOR correlations with metabolites were examined only within the ASD group as the TD group did not have significant symptoms or variability in SOR. Thalamic GABA+/Cr was significantly negatively correlated with SOR after controlling for volume-fraction gray matter and SCARED (*r* = −0.48, *n* = 28, *p* < 0.05, Fig. 1A), indicating that ASD youth with higher SOR had lower thalamic GABA+/Cr. Thalamic GABA+/Glx was also correlated with SOR (*r* = −0.42, *n* = 28, *p* < 0.05) and this correlation was driven by the GABA+/Cr values. SSC Glx/Cr was significantly positively correlated with SOR (*r* = 0.68, *n* = 20, *p* < 0.01, Fig. 1B), indicating that ASD youth with higher SOR had higher SSC Glx/Cr (Fig. 1B). Thalamic Glx/Cr, SSC GABA+/Cr, and SSC GABA+/Glx were not significantly correlated with SOR and the Pearson’s *r* coefficients of correlation with SOR were significantly different for thalamic GABA+/Cr compared to SSC GABA+/Cr (*p* < 0.01) and for thalamic Glx/Cr compared to SSC Glx/Cr (*p* < 0.001). Additionally, there were no significant differences between correlations of either thalamic GABA+/Cr or SSC Glx/Cr with SOR between those youth taking and not taking psychotropic medication. There were 14 ASD youth with adequate MRS data from both the thalamus and SSC which demonstrated a trend toward a relationship of lower thalamic GABA + /Cr with higher SSC Glx/Cr controlling for volume-fraction gray matter (*r* = −0.394, *p* = 0.060; eFig. 2).

### Relationship between thalamic neurometabolites and thalamic intrinsic functional connectivity

**Fig. 2:**
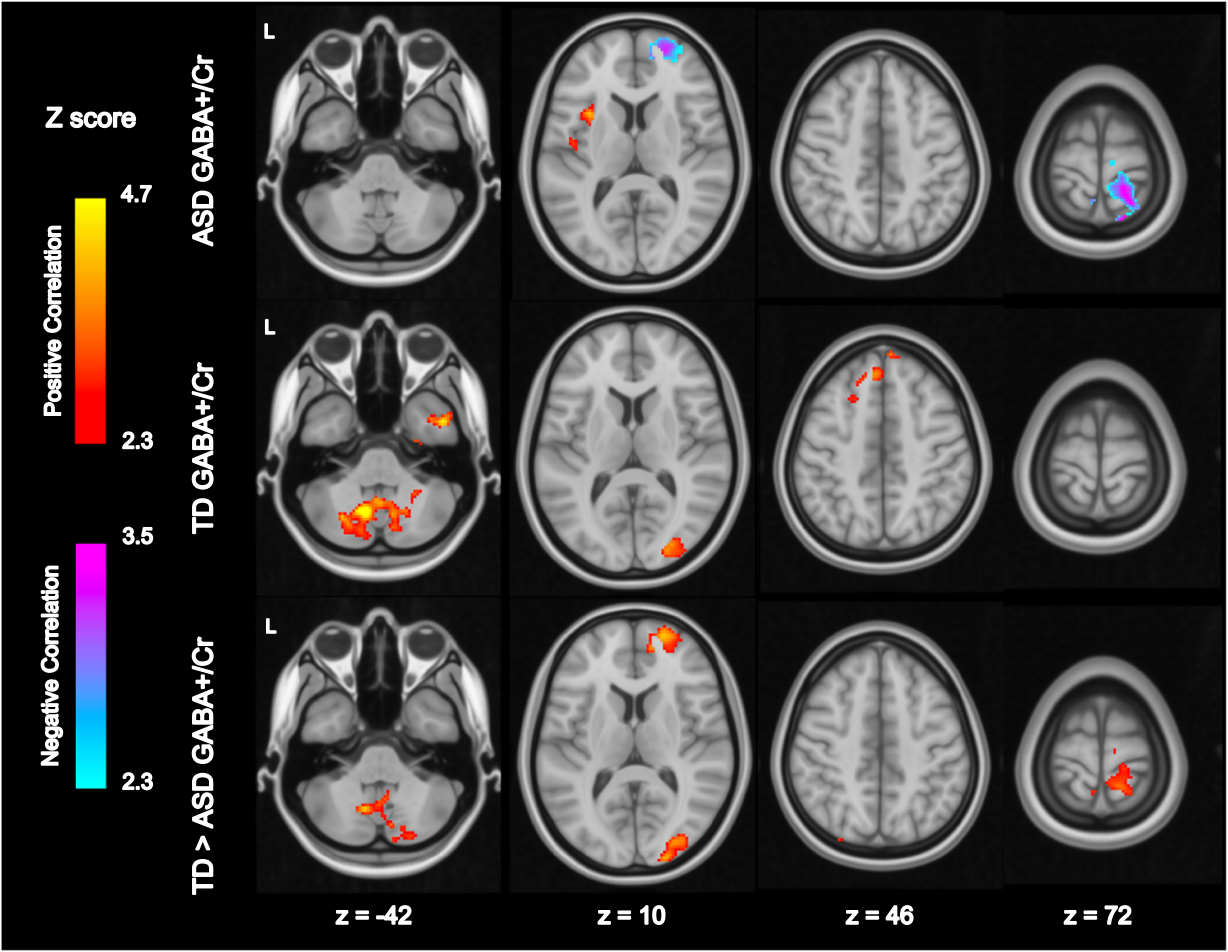
Thalamus Functional Connectivity Related to Thalamic GABA+/Cr. Whole-brain resting-state analyses using bilateral thalamus seed with GABA+/Cr as a bottom-up regressor. Within-group and between-group contrasts are thresholded at *Z* > 2.30 and cluster corrected at *p* < 0.05 (see eFig. 3.A for unthresholded maps). ASD Autism spectrum disorders, L Left, TD Typically developing.

Within the ASD group, thalamic GABA+/Cr was positively correlated with functional connectivity between bilateral thalamus and left insula, and negatively correlated with connectivity between thalamus and: (1) right SSC and (2) right anterior prefrontal cortex (anterior-PFC). Within the TD group, thalamic GABA+/Cr was positively correlated with connectivity between the thalamus and: 1) right occipital cortex, (2) cerebellum, (3) left superior frontal gyrus, and (4) right inferior temporal gyrus. A between-groups comparison further showed that thalamic GABA+/Cr was more negatively correlated with functional connectivity (i.e. higher GABA+/Cr predicted lower connectivity) in ASD than TD between bilateral thalamus and (1) right SSC, (2) right occipital cortex, (3) right anterior-PFC, and (4) cerebellum (Fig. 2, eFig. 3.A and eTable 4). There were no significant correlations between thalamic Glx and thalamic connectivity. To illustrate the relationship of neurometabolites with resting-state connectivity by diagnostic groups, thalamic GABA+/Cr was median split into low and high categories using all subjects. Thalamic connectivity differences between groups were accounted for by the low GABA+/Cr category (eFig. 4), suggesting that ASD youth with low but not high thalamic GABA+/Cr show atypical thalamic connectivity. Within the TD group, there were no differences in age, SOR, autism severity, anxiety, IQ, or rsfMRI motion measures between categories. Within the ASD group, the low GABA category had higher SOR and greater absolute motion (eTable 5) compared to the high GABA group, so absolute motion was tested as a covariate in these comparisons but it did not alter the significant differences reported.

### Relationship between SSC neurometabolites and SSC intrinsic functional connectivity

**Fig. 3:**
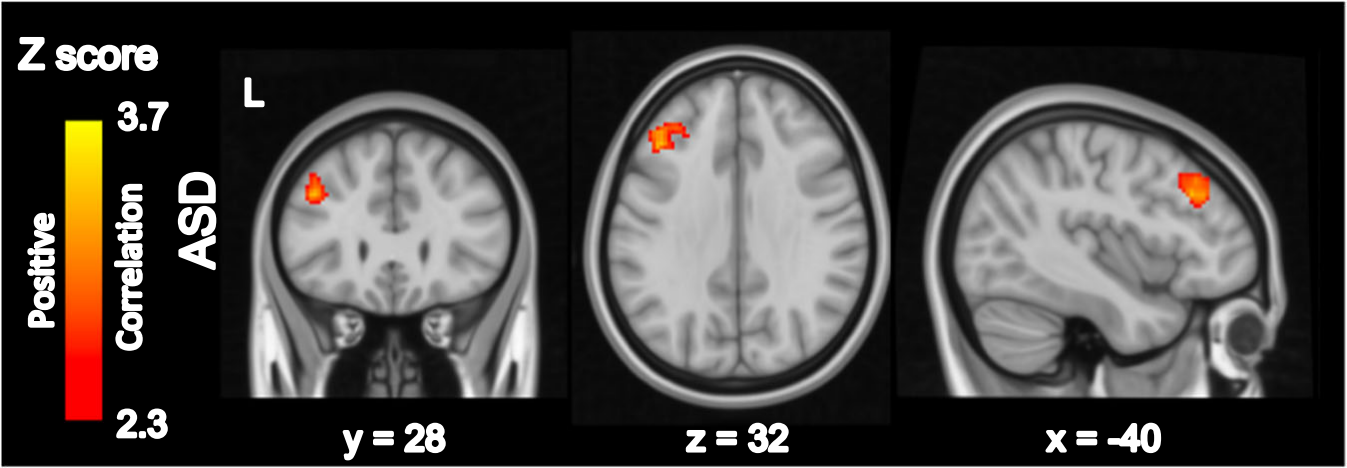
Somatosensory Cortex Connectivity Related to SSC Glx/Cr. Whole-brain resting-state analysis using bilateral precentral gyrus seed with Glx/Cr as a bottom-up regressor. Within-group and between-group contrasts were thresholded at *Z* > 2.30 and cluster corrected at *p* < 0.05 (see eFig. 3.B for unthresholded maps), only within ASD demonstrated a significant relationship with Glu/Cr. ASD Autism spectrum disorders, L Left.

Within the ASD group, SSC Glx/Cr correlated with greater resting-state functional connectivity between the bilateral SSC and left middle frontal gyrus (Fig. 3, eFig. 3.B, eTable 4). There were no significant correlations between SSC connectivity and Glx/Cr within the TD group or significant differences between groups. SSC GABA+/Cr did not correlate with SSC connectivity. As above, when SSC Glx was median split into low and high categories, SSC connectivity differences between groups were accounted for by the high Glx/Cr category, suggesting that ASD youth with high but not low SSC Glx/Cr show atypical SSC connectivity (eFig. 4H). Within both the ASD and TD groups, there were no differences in age, SOR, autism severity, anxiety, IQ, or rsfMRI motion measures between categories (eTable 6).

### Relationship between intrinsic functional connectivity measures and SOR

Parameter estimates of connectivity strength were extracted from regions where significant correlations between neurometabolites and connectivity were observed as reported above, to determine whether connectivity in these regions also correlated with SOR. Strength of functional connectivity between the thalamus and left insular cortex was negatively correlated with SOR (*r* = −0.41, *p* < 0.05, eFig. 5.A). SOR was positively correlated with strength of connectivity between thalamus-right anterior-PFC (*r* = 0.47, *p* < 0.05, eFig. 5.B), thalamus-cerebellum (*r* = 0.49, *p* < 0.01) and SSC-left middle frontal gyrus (*r* = 0.41, *p* < 0.05). Connectivity between the thalamus and other regions, including right SSC and right occipital pole, were not significantly correlated with SOR.

## Discussion

Using MR spectroscopy, we found that, in ASD, reduced thalamic GABA was significantly related to both behavioral symptoms of SOR as well as atypical thalamic connectivity with sensory processing regions. As a whole, our results suggest that for ASD youth, abnormalities of the thalamic neurochemical balance could interfere with the thalamic role in responsively and flexibly modulating attention to sensory information.

To our knowledge, this is the first report of a significant relationship between in vivo thalamic GABA and SOR as well as the first examination of thalamic GABA in ASD youth. Our finding that lower GABA concentration in the thalamus was correlated with higher SOR symptoms in youth with ASD supports the role of the thalamus as the sensory gatekeeper and thalamic GABA as a critical component of sensory modulation. While the thalamus is a relay station for sensory information coming from the periphery, it also exerts control over many higher-level processes including functional interactions between cortical areas^59^. For youth with ASD and SOR, decreased thalamic GABA may impair their ability to responsively attend to and filter sensory stimuli based on changing task-related demands or state-dependent drives. This impairment could underlie their atypical aversive experiences and increased salience of extraneous stimuli at the expense of developmentally appropriate attention to socially relevant stimuli.

Our finding of somatosensory cortex Glx/Cr correlation with SOR in ASD demonstrates greater cortical excitatory tone associated with higher SOR and is consistent with the E/I imbalance model of sensory dysregulation^21,23^. The lack of significant correlation between SSC GABA+/Cr and SOR in our group suggests that GABAergic regulation of sensory responsivity is more specific to the thalamus, highlighting its central modulatory role. Previous studies of cortical GABA+ have demonstrated relationships between SSC GABA+ concentration and sensory discrimination^29,32,34,60^, and support theories of insufficient cortical GABA causing hyper-reactivity during key developmental stages leading to inadequate sensory system tuning at the level of cortical minicolumns^22–24^. In addition to these potentially early-developing bottom-up sensory system alterations in discrimination or sensitivity, our findings suggest that SOR involves deficits in top-down attentional and behavioral regulation processes governed by the thalamus, later in development.

We additionally hypothesized that if thalamic GABA underlies SOR, it should also modulate thalamic circuits implicated in SOR. Prior studies have demonstrated atypical thalamocortical connectivity in ASD with recurring themes of thalamic hyperconnectivity with primary sensory and motor regions, and hypoconnectivity with frontal and other supramodal cortical regions^61–67^. Our resting-state fMRI analyses elucidated several atypical relationships between thalamic GABA+/Cr and thalamic connectivity in ASD compared to TD youth, especially in regions related to sensory and salience processing. In particular, only ASD youth with lower GABA displayed atypical thalamic connectivity with somatosensory and occipital cortices, suggesting a model wherein high GABA may actually compensate for deficits in sensory reactivity (at least at this relatively later stage of childhood). This model is consistent with our findings of no group differences in thalamic GABA+/Cr despite large differences in SOR.

Thalamic engagement in processes such as perception, arousal, attentional selection, and sensory regulation^68–70^ requires complex GABAergic inhibitory control accomplished via two primary systems: (1) a bottom-up system that broadly controls signal strength through the thalamic reticular nucleus (TRN) that is important for stimulus-and emotion-driven attention and decision making; and (2) a top-down system that more specifically affects information content via precise spike-timing facilitation by extrathalamic inhibitory neurons and supports plan-driven attentional focus^12,71^. Notably, the amygdala projects to TRN to facilitate selective attention guided by emotions and drives through TRN-mediated lateral inhibition of alternative cortico-thalamic loops^71,72^. In ASD, SOR is associated with increases in functional connectivity between amygdala and the pulvinar nucleus of the thalamus when responding to aversive sensory stimulation^15^. Our finding of SOR relating to reduced thalamic GABA could indicate atypical functioning of either of these systems. However, the relationship between GABA and atypical thalamic functional connectivity with sensory cortices is likely more indicative of increased feedforward signal in the bottom-up system. In contrast, our finding that within the ASD group, decreased thalamic GABA was associated with thalamic *hypoconnectivity* with the insula, is more in line with atypical top-down thalamic function. The insula, a salience network hub, plays a role in flexible, context-dependent attentional focus^73^. Furthermore, thalamic-insula but not thalamic-sensory cortex connectivity was correlated with SOR, a measure of behavioral sensory reactivity thought to reflect reduced top-down regulation of sensory responses in ASD^74^. In other words, while atypical thalamic-sensory connectivity might reflect aberrant sensory processing, only deficits in top-down regulatory processes would result in the abnormal behavioral output associated with SOR.

These interpretations of the relationships between ^1^H-MRS in vivo GABA measurements and thalamocortical circuitry are preliminary as ^1^H-MRS necessarily has limited spatial and temporal resolution. Also, we report GABA+/Cr and Glx/Cr rather than absolute levels of GABA and Glu, although Cr levels did not differ between groups (Table 2). In our case, we were further limited by the smaller head size of children which made it difficult to fit a large enough acquisition volume for acceptable signal-to-noise ratio while focusing on the regions of interest. Additionally, our relatively small sample size of subjects with acceptable ^1^H-MR spectra from both thalamus and SSC (14 ASD) limited our power to assess for metabolite relationships between these regions of interest and suggests that the trend toward a relationship of lower thalamic GABA+/Cr with higher SSC Glx/Cr seen here should be replicated in a larger sample. Animal models that are able to utilize causal, circuit-specific techniques will be required to determine the direction and specificity of effects. Nonetheless, our study builds on previous evidence of GABA system atypicalities at the mechanistic levels of genetic, protein expression, and cellular pathophysiology^75–77^ to show that GABA-mediation of thalamocortical circuits is a key component of SOR in ASD.

While research on sensory over-responsivity in ASD and other disorders is still in its infancy, our results showing a relationship between thalamic inhibitory tone, SOR, and thalamic intrinsic connectivity support current theories that SOR is related to deficits in top-down sensory regulation^13,15,74^, and further specifies thalamic inhibition as a likely important mechanism of such top-down modulation. Given the functional impairment associated with SOR, its treatment is likely to result in improvements in related deficits such as self-care, socialization skills, and anxiety, consistent with emerging research findings^5^. Identifying specific neurobiological mechanisms underlying sensory over-responsivity, such as the reduced thalamic GABA found here, can inform potential treatment targets and lead to more effective interventions.

## Supplementary information

Supplemental Material
