# Supplementary material for: Sensory over-responsivity is related to GABAergic inhibition in thalamocortical circuits
Source: Transl Psychiatry. 2021 Jan 12;11:39. doi: 10.1038/s41398-020-01154-0 (PMC7804323; doi:10.1038/s41398-020-01154-0)

#

**Supplemental Content**

[eTable 1. 1H-MRS and fMRI Quality Assurance 2](#_Toc52180679)

[eTable 2. Age-matched demographics and behavioral measures 3](#_Toc52180680)

[eTable 3. Medications 3](#_Toc52180681)

[eTable 4. Montreal Neurological Coordinates for Resting State fMRI Within and Between Group Comparisons 4](#_Toc52180682)

[eTable 5. Demographics, behavioral measures and rsfMRI motion for Thalamic GABA+/Cr median split groups 5](#_Toc52180683)

[eTable 6. Demographics, behavioral measures and rsfMRI motion for SSC Glx/Cr median split groups 6](#_Toc52180684)

[eFigure 1. Edited ^1^H-MRS Example Spectra and Voxel Localization 7](#_Toc52180685)

[eFigure 2. Relationship between thalamic GABA+/Cr and SMS Glx/Cr 8](#_Toc52180686)

[eFigure 3. Thalamus Functional Connectivity Related to Thalamic GABA+/Cr. 9](#_Toc52180687)

[eFigure 4. Resting State Connectivity by Low/High Metabolite Level 10](#_Toc52180688)

[eFigure 5. Thalamocortical Connectivity Related to Sensory Over-Responsivity Severity 11](#_Toc52180689)

**Title:**

Sensory Over-Responsivity is Related to GABAergic Inhibition in Thalamocortical Circuits

**Authors:**

E. Turner Wood, MD, PhD^1,2,^ Kaitlin K. Cummings, BS^1^, Jiwon Jung, BS^1^, Genevieve Patterson, BS^1^, Nana Okada, BA^1^, Jia Guo, PhD^3^, Joseph O'Neill, PhD^2^, Mirella Dapretto, PhD ^1^, Susan Y. Bookheimer, PhD ^1^, S. A. Green, PhD^1^

1. Dept of Psychiatry and Biobehavioral Sciences, University of California, Los Angeles, Los Angeles, CA
2. Division of Child & Adolescent Psychiatry, UCLA Jane & Terry Semel Institute for Neuroscience, Los Angeles, CA
3. Department of Psychiatry & The Zuckerman Institute, Columbia University, New York, NY

**Corresponding Author:**

S. A. Green, Ph.D.

Address: 660 Charles E. Young Drive South, Los Angeles, CA 90095

Phone: 310-983-3667

Email: [SAGreen@mednet.ucla.edu](mailto:SAGreen@mednet.ucla.edu)

|  | **ASD**  (mean ± SD) | **TD**  (mean ± SD) | *t*-test of means | *F* test of variances |
| --- | --- | --- | --- | --- |
| Subjects with useable Thalamus ^1^H-MRS (n)^a^ | 29 | 31 |  |  |
| Thalamus Voxel Tissue Segmentation |  |  |  |  |
| Fraction GM | 0.39 ± 0.09 | 0.43 ± 0.07 | *t* = -1.95,  *p* = 0.056 | *F* = 0.88,  *p* = 0.35 |
| Fraction WM | 0.53 ± 0.09 | 0.50 ± 0.09 | *t* = 1.36,  *p* = 0.18 | *F* < 0.01,  *p* = 0.98 |
| Fraction CSF | 0.08 ± 0.03 | 0.07 ± 0.03 | *t* = 1.11,  *p* = 0.27 | *F* = 0.88,  *p* = 0.35 |
| Subjects with useable Right SS Cortex ^1^H-MRS (n)^b^ | 21 | 23 |  |  |
| Right SS Cortex Voxel Tissue Segmentation |  |  |  |  |
| Fraction GM | 0.44 ± 0.05 | 0.47 ± 0.06 | *t* = -1.49,  *p* = 0.14 | *F* = 1.06,  *p* = 0.31 |
| Fraction WM | 0.51 ± 0.07 | 0.47 ± 0.09 | *t* = 1.58,  *p* = 0.12 | *F* = 2.07,  *p* = 0.16 |
| Fraction CSF | 0.05 ± 0.02 | 0.06 ± 0.04 | *t* = -1.13,  *p* = 0.26 | *F* = 4.11,  *p* = 0.049 |
| Subjects with useable Resting State fMRI (n) | 34 | 29 |  |  |
| Resting State Scan Motion |  |  |  |  |
| Maximum Absolute Motion (mm) | 1.41 ± 0.81 | 1.24 ± 0.70 | *t* = 0.87,  *p* = 0.39 | *F* = 0.93,  *p* = 0.34 |
| Mean Absolute Motion (mm) | 0.52 ± 0.28 | 0.44 ± 0.22 | *t* = 1.24,  *p* = 0.22 | *F* = 0.85,  *p* = 0.36 |
| Maximum Relative Motion (mm) | 0.96 ± 0.91 | 0.73 ± 0.59 | *t* = 1.15,  *p* = 0.25 | *F* = 1.44,  *p* = 0.23 |
| Mean Relative Motion (mm) | 0.14 ± 0.07 | 0.12 ± 0.05 | *t* = 1.58,  *p* = 0.12 | *F* = 2.80,  *p* = 0.10 |
| ICA-AROMA |  |  |  |  |
| Total Number Components | 257.5 ± 33.9 | 263.8 ± 20.2 | *t* = -0.88,  *p* = 0.39 | *F* = 0.96,  *p* = 0.33 |
| Percent Components Kept (%) | 57 ± 11 | 56 ± 10 | *t* = 0.42,  *p* = 0.68 | *F* = 0.25,  *p* = 0.62 |
| 1. 6 ASD and 2 TD subjects had unacceptable Thalamus MRS data due to poor SNR and/or poor goodness of fit. Demographic and behavioral measures for this sub-group were not substantially different from the full group. 2. Due to study timing, only 21 ASD and 24 TD SSC voxel MRS data were acquired. 1 TD has unacceptable SSC MRS data due to poor SNR and poor goodness of fit. Demographic and behavioral measures for this sub-group were not substantially different from the full group with the exception of age (ASD mean = 13.5, TD mean = 12.7, *ns*). | | | | |

eTable 1. 1H-MRS and fMRI Quality Assurance

eTable 2. Age-matched demographics and behavioral measures

|  | **ASD**  (mean ± SD) | **TD**  (mean ± SD) | ***t*** or **χ^2^** |
| --- | --- | --- | --- |
| ***Subjects*** (number) | 29 | 29 |  |
| ***Sex*** (females) | 8 | 11 | *p*=0.58 |
| ***Age (years)*** | 13.90 ± 2.5 | 13.74 ± 2.6 | *p*=0.82 |
| ***Social Responsivity Scale*** (SRS) Total | 78.79 ± 26.5 | 19.93 ± 18.2 | *p*<0.001 |
| ***Anxiety Total Score*** (SCARED Parent) | 18.62 ± 11.3 | 6.38 ± 6.7 | *p*<0.001 |
| ***WASI Full-scale IQ*** | 106.00 ± 15.5 | 116.00 ± 13.2 | *p*=0.01 |
| *WASI Verbal IQ* | 101.69 ± 17.5 | 115.48 ± 12.9 | *p*<0.01 |
| *WASI Performance IQ* | 110.00 ± 16.8 | 112.86 ± 13.2 | *p*=0.47 |
| ***SOR Total Score*** (SenSOR Parent) | 74.97 ± 25.6 | 49.07 ± 6.8 | *p*<0.001 |

SCARED = Screen for Child Anxiety Related Emotional Disorders; SenSOR = Sensory Over-Responsivity Inventory

eTable 3. Medications

|  | ASD (n) | TD (n) |
| --- | --- | --- |
| **No Meds** | 18 | 27 |
| **Vitamin/Supplement** | 7 | 5 |
| **SSRI** | 8 | 0 |
| **Stimulant** | 8 | 0 |
| **α_2_ Agonist** | 4 | 0 |
| **Antipsychotic** | 4 | 0 |
| **Antihistamine** | 3 | 0 |

| eTable 4. Montreal Neurological Coordinates for Resting State fMRI Within and Between Group Comparisons | | | | | | | | | | | | | | | | | | | | | | | | | | | | | | | | | | |
| --- | --- | --- | --- | --- | --- | --- | --- | --- | --- | --- | --- | --- | --- | --- | --- | --- | --- | --- | --- | --- | --- | --- | --- | --- | --- | --- | --- | --- | --- | --- | --- | --- | --- | --- |
|  | **ASD +** | | | | | | | | **ASD -** | | | | | | | | | **TD +** | | | | | | | | **TD > ASD** | | | | | | | | |
|  | **Voxels** | **Max *Z*** | **MNI Peak (mm)** | | | | | | **Voxels** | | **Max *Z*** | | **MNI Peak (mm)** | | | | | **Voxels** | | **Max *Z*** | | **MNI Peak  (mm)** | | | | **Voxels** | | **Max *Z*** | | **MNI Peak (mm)** | | | | |
|  |  |  | **x** | | **y** | | **z** | |  |  |  |  | **x** | **y** | | **z** | |  |  |  |  | **x** | **y** | | **z** |  |  |  |  | **x** | | **y** | **z** | |
| ***Thalamus GABA+/Cr with:*** | | | |  | |  | |  | |  | |  | | |  | |  | |  | |  | | |  | | |  | |  | |  | | |  |
| Left Insular Cortex | 421 | 4.2 | -42 | | -6 | | 0 | |  | |  | |  |  | |  | |  | |  | |  |  | |  |  | |  | |  | |  |  | |
| Right Inferior Frontopolar Gyrus (anterior PFC) |  |  |  | |  | |  | | 522 | | 3.52 | | 22 | 54 | | 4 | |  | |  | |  |  | |  | 633 | | 3.74 | | 22 | | 54 | 4 | |
| Right Precentral Gyrus |  |  |  | |  | |  | | 684 | | 3.78 | | 8 | -24 | | 78 | |  | |  | |  |  | |  | 522 | | 3.69 | | 10 | | -22 | 78 | |
| *Right Postcentral Gyrus* |  |  |  | |  | |  | |  | | *3.76* | | *16* | *-38* | | *76* | |  | |  | |  |  | |  |  | | *3.11* | | *14* | | *-46* | *70* | |
| Right Occipital Gyrus |  |  |  | |  | |  | |  | |  | |  |  | |  | | 643 | | 4.14 | | 42 | -86 | | -14 | 420 | | 3.66 | | 30 | | -94 | 16 | |
| Cerebellum |  |  |  | |  | |  | |  | |  | |  |  | |  | | 1368 | | 4.86 | | -10 | -62 | | -42 | 843 | | 4.52 | | 12 | | -74 | -34 | |
| Left Sup Frontal Gyrus |  |  |  | |  | |  | |  | |  | |  |  | |  | | 309 | | 3.29 | | -6 | 38 | | 44 |  | |  | |  | |  |  | |
| Right Inferior Temporal Gyrus |  |  |  | |  | |  | |  | |  | |  |  | |  | | 571 | | 4.77 | | 44 | 0 | | -36 |  | |  | |  | |  |  | |
| ***SSC Glx/Cr with:*** |  |  |  | |  | |  | |  | |  | |  |  | |  | |  | |  | |  |  | |  |  | |  | |  | |  |  | |
| Left Middle Frontal Gyrus | 298 | 3.34 | -40 | | 28 | | 32 | |  | |  | |  |  | |  | |  | |  | |  |  | |  |  | |  | |  | |  |  | |
| Note: ASD+, ASD- and TD+ coordinates indicate clusters with significant within-group positive or negative functional connectivity; between-group TD > ASD coordinates indicate clusters where the TD group has significant greater connectivity than ASD. x, y, and z refer to the left–right, anterior–posterior, and inferior–superior dimensions, respectively; *Z* refers to the *Z*-score at those coordinates (local maxima or *submaxima*). For within- and between-group analyses, voxels were thresholded at *Z* > 2.3 (*p*<0.01), and cluster corrected for multiple comparisons (*p*<0.05). | | | | | | | | | | | | | | | | | | | | | | | | | | | | | | | | | | |

eTable 5. Demographics, behavioral measures and rsfMRI motion for Thalamic GABA+/Cr median split groups

|  | **ASD** | | | | **TD** | | | |
| --- | --- | --- | --- | --- | --- | --- | --- | --- |
|  | Low GABA (mean ± SD)  n = 12 | High GABA (mean ± SD)  n = 16 | Levene's Test for Equality of Variances | t-test for Equality of Means | Low GABA (mean ± SD)  n = 16 | High GABA (mean ± SD)  n = 12 | Levene's Test for Equality of Variances | t-test for Equality of Means |
| **Age** (years) | 15.20 | 15.018 | *F*=.30 | *t*=.18 | 13.02 | 13.52 | *F*=6.51 | *t*=-.47^&^ |
|  | ± 2.54 | ± 2.727 | *p*=.589 | *p*=.86 | ± 3.40 | ± 2.33 | *p*=.017* | *p*=.65 |
| ***SOR Total Score*** (SenSOR Parent) | 85.17 | 64.63 | *F*=.88 | *t*=2.39 | 49.69 | 48.33 | *F*=3.40 | *t*=.52 |
|  | ± 27.06 | ± 18.518 | *p*=.36 | *p*=.025* | ± 8.41 | ± 3.92 | *p*=.08 | *p*=.61 |
| ***Social Responsivity Scale*** (SRS) Total | 80.67 | 71.63 | *F*=.60 | *t*=.81 | 16.94 | 19.50 | *F*=.03 | *t*=-.40 |
|  | ± 29.19 | ± 29.529 | *p*=.45 | *p*=.43 | ± 15.22 | ± 18.63 | *p*=.87 | *p*=.69 |
| ***Anxiety Total Score*** (SCARED Parent) | 19.50 | 17.19 | *F*=.11 | *t*=.47 | 44.19 | 44.42 | *F*=.02 | *t*=-.09 |
|  | ± 12.12 | ± 13.343 | *p*=.74 | *p*=.64 | ± 6.36 | ± 7.54 | *p*=.90 | *p*=.93 |
| ***WASI Full-scale IQ*** | 105.33 | 107.19 | *F*=.62 | *t*=-.34 | 115.31 | 120.00 | *F*=.19 | *t*=-1.02 |
|  | ± 15.96 | ± 13.253 | *p*=.44 | *p*=.74 | ± 12.77 | ± 10.89 | *p*=.67 | *p*=.32 |
| **Maximum Absolute Motion** (mm) | 1.78 | 1.19 | *F*=4.81 | *t*=2.21^&^ | 1.32 | 1.21 | *F*=.27 | *t*=.39 |
|  | ± .79 | ± .54 | *p*=.037* | *p*=.040* | ± .80 | ± .59 | *p*=.61 | *p*=.70 |
| **Mean Absolute Motion** (mm) | .72 | .43 | *F*=.99 | *t*=3.06 | .49 | .40 | *F*=.23 | *t*=.96 |
|  | ± .30 | ± .22 | *p*=.33 | *p*=.005* | ± .21 | ± .24 | *p*=.64 | *p*=.35 |
| **Maximum Relative Motion** (mm) | 1.07 | .80 | *F*=1.22 | *t*=.85 | .70 | .81 | *F*=.44 | *t*=-.46 |
|  | ± 1.13 | ± .51 | *p*=.28 | *p*=.40 | ± .73 | ± .39 | *p*=.52 | *p*=.65 |
| **Mean Relative Motion** (mm) | .14 | .14 | *F*=1.65 | *t*=.05 | .13 | .11 | *F*=1.80 | *t*=.997 |
|  | ± .08 | ± .06 | *p*=.21 | *p*=.96 | ± .06 | ± .04 | *p*=.19 | *p*=.33 |
| **ICA Total Number Components** | 245.33 | 263.75 | *F*=4.91 | *t*=-1.20^&^ | 267.63 | 253.36 | *F*=.26 | *t*=2.00 |
|  | ± 50.40 | ± 18.88 | *p*=.036* | *p*=.25 | ± 17.98 | ± 18.58 | *p*=.62 | *p*=.057 |
| **ICA Components Kept** (%) | 55.55 | 58.19 | *F*=.12 | *t*=-.74 | 53.75 | 58.27 | *F*=.91 | *t*=-1.14 |
|  | ± 9.39 | ± 9.45 | *p*=.73 | *p*=.47 | ± 10.64 | ± 9.22 | *p*=.35 | *p*=.26 |

^&^ equal variance not assumed; * p<0.0

eTable 6. Demographics, behavioral measures and rsfMRI motion for SSC Glx/Cr median split groups

|  | **ASD** | | | | **TD** | | | |
| --- | --- | --- | --- | --- | --- | --- | --- | --- |
|  | Low Glx (mean ± SD)  n = 8 | High Glx (mean ± SD)  n = 12 | Levene's Test for Equality of Variances | t-test for Equality of Means | Low Glx (mean ± SD)  n = 10 | High Glx (mean ± SD)  n = 10 | Levene's Test for Equality of Variances | t-test for Equality of Means |
| **Age** (years) | 12.55 | 14.05 | *F*=2.85 | *t*=-1.31 | 12.62 | 13.60 | *F*=2.06 | *t*=-.73 |
|  | ± 2.08 | ± 2.74 | *p*=.11 | *p*=.21 | ± 3.33 | ± 2.67 | *p*=.17 | *p*=.48 |
| ***SOR Total Score*** (SenSOR Parent) | 70.00 | 84.50 | *F*=7.57 | *t*=-1.39^&^ | 46.60 | 50.10 | *F*=4.99 | *t*=-1.56^&^ |
|  | ± 11.45 | ± 33.30 | *p*=.013* | *p*=.19 | ± 3.27 | ± 6.28 | *p*=.038* | *p*=.14 |
| ***Social Responsivity Scale*** (SRS) Total | 82.25 | 75.25 | *F*=.21 | *t*=.55 | 19.30 | 28.60 | *F*=1.80 | *t*=-1.01 |
|  | ± 31.24 | ± 25.87 | *p*=.65 | *p*=.59 | ± 16.73 | ± 23.87 | *p*=.20 | *p*=.33 |
| ***Anxiety Total Score*** (SCARED Parent) | 22.38 | 18.92 | *F*=.002 | *t*=.61 | 44.80 | 48.40 | *F*=2.17 | *t*=-.91 |
|  | ± 13.45 | ± 11.63 | *p*=.97 | *p*=.55 | ± 7.16 | ± 10.27 | *p*=.16 | *p*=.38 |
| ***WASI Full-scale IQ*** | 108.13 | 102.08 | *F*=1.23 | *t*=.75 | 119.10 | 114.90 | *F*=1.89 | *t*=.63 |
|  | ± 21.63 | ± 14.77 | *p*=.28 | *p*=.47 | ± 9.11 | ± 19.16 | *p*=.19 | *p*=.54 |
| **Maximum Absolute Motion** (mm) | 1.42 | 1.32 | *F*=.03 | *t*=.25 | 1.50 | 1.21 | *F*=.21 | *t*=.81 |
|  | ± 1.012 | ±.77 | *p*=.86 | *p*=.81 | ± .81 | ± .75 | *p*=.66 | *p*=.43 |
| **Mean Absolute Motion** (mm) | .50 | .58 | *F*=1.66 | *t*=-.57 | .47 | .42 | *F*=.28 | *t*=.47 |
|  | ±. .22 | ± .38 | *p*=.21 | *p*=.57 | ± .18 | ± .25 | *p*=.60 | *p*=.64 |
| **Maximum Relative Motion** (mm) | 1.12 | .69 | *F*=3.07 | *t*=1.25 | .95 | .72 | *F*=.80 | *t*=.74 |
|  | ± 1.08 | ± .40 | *p*=.10 | *p*=.23 | ± .86 | ± .38 | *p*=.39 | *p*=.47 |
| **Mean Relative Motion** (mm) | .15 | .15 | *F*=1.34 | *t*=-.18 | .15 | .11 | *F*=1.84 | *t*=1.86 |
|  | ± .06 | ± .08 | *p*=.26 | *p*=.86 | ± .06 | ± .04 | *p*=.19 | *p*=.09 |
| **ICA Total Number Components** | 282.63 | 243.25 | *F*=.68 | *t*=2.31 | 269.80 | 266.00 | *F*=1.20 | *t*=.36 |
|  | ± 23.06 | ± 44.18 | *p*=.42 | *p*=.033* | ± 21.17 | ± 25.27 | *p*=.29 | *p*=.73 |
| **ICA Components Kept** (%) | 61.44 | 54.61 | *F*=.50 | *t*=1.19 | .53 | .55 | *F*=.07 | *t*=-.32 |
|  | ± 10.28 | ± 13.81 | *p*=.49 | *p*=.25 | ± .10 | ± .10 | *p*=.80 | *p*=.76 |

^&^ equal variance not assumed; * p<0.05

eFigure 1. Edited ^1^H-MRS Example Spectra and Voxel Localization

Exemplary spectra and voxel locations are shown for edited ^1^H-MRS spectra acquired from (A) bilateral thalamus (volume = FH 25mm × LR 35mm × AP 10mm = 8750mm^2^); and (B) right somatosensory cortex (volume = 20mm × 30mm × 20mm = 12000mm^2^).


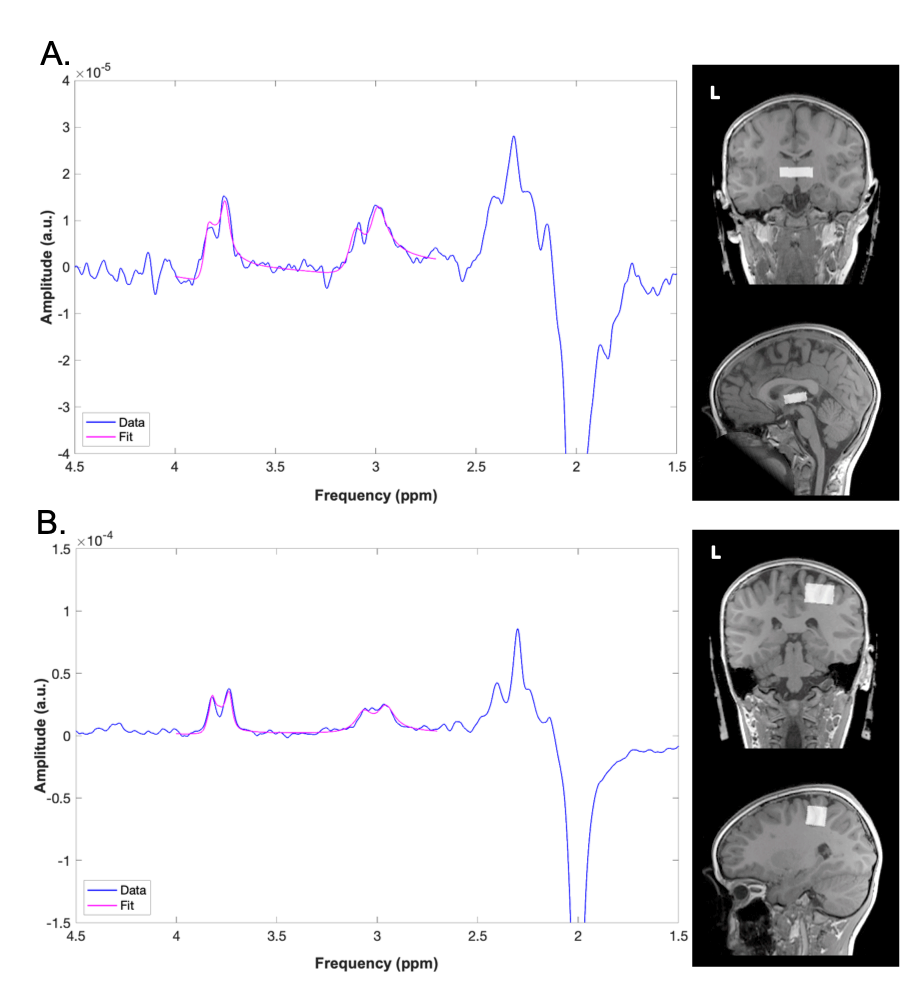


eFigure 2. Relationship between thalamic GABA+/Cr and SMS Glx/Cr

There was a trend toward a negative correlation between thalamic GABA+/Cr and SSC Glx/Cr controlling for volume-fraction gray matter (*r* =-0.39, *p*=0.06, n = 14).


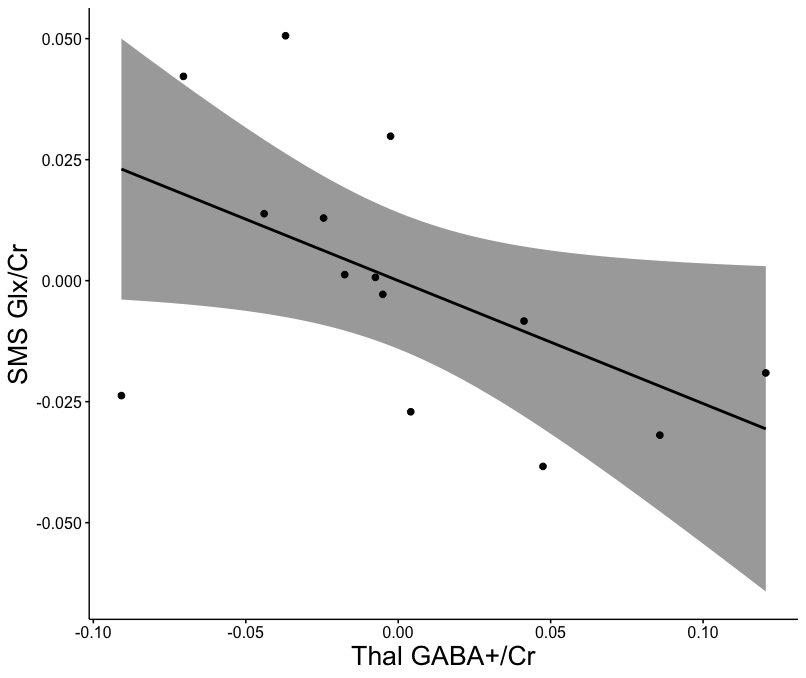


eFigure 3. Thalamus Functional Connectivity Related to Thalamic GABA+/Cr.

1. Whole-brain resting-state analyses using bilateral thalamus seed with GABA+/Cr as a bottom-up regressor. Within-group and between-group contrasts without cluster defining threshold (See main article Figure 2 for thresholded maps).
2. Whole-brain resting state analysis using bilateral precentral gyrus seed with Glx/Cr as a bottom-up regressor. Within-group and between-group contrasts without cluster defining threshold (See main article Figure 3 for thresholded maps). ASD = autism spectrum disorders, L=left, TD=typically developing.

Z score

4.5

-4.5


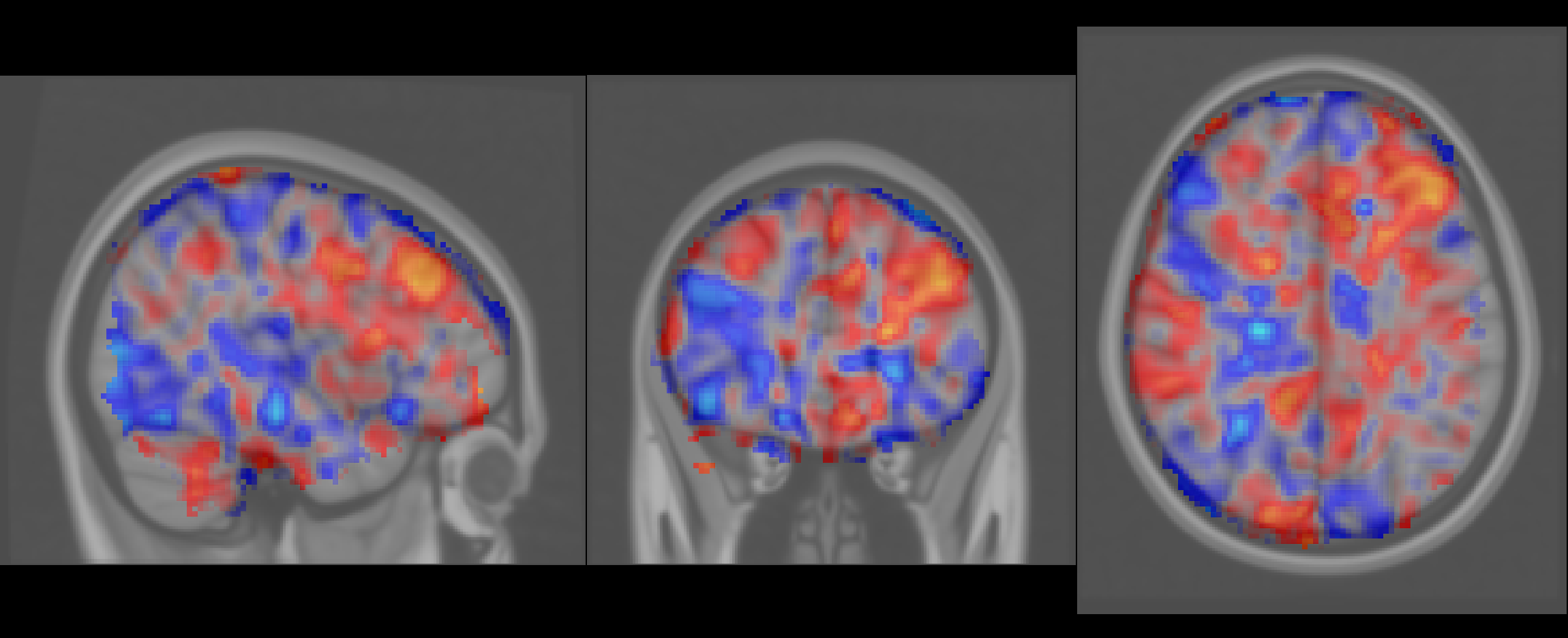

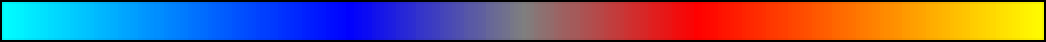

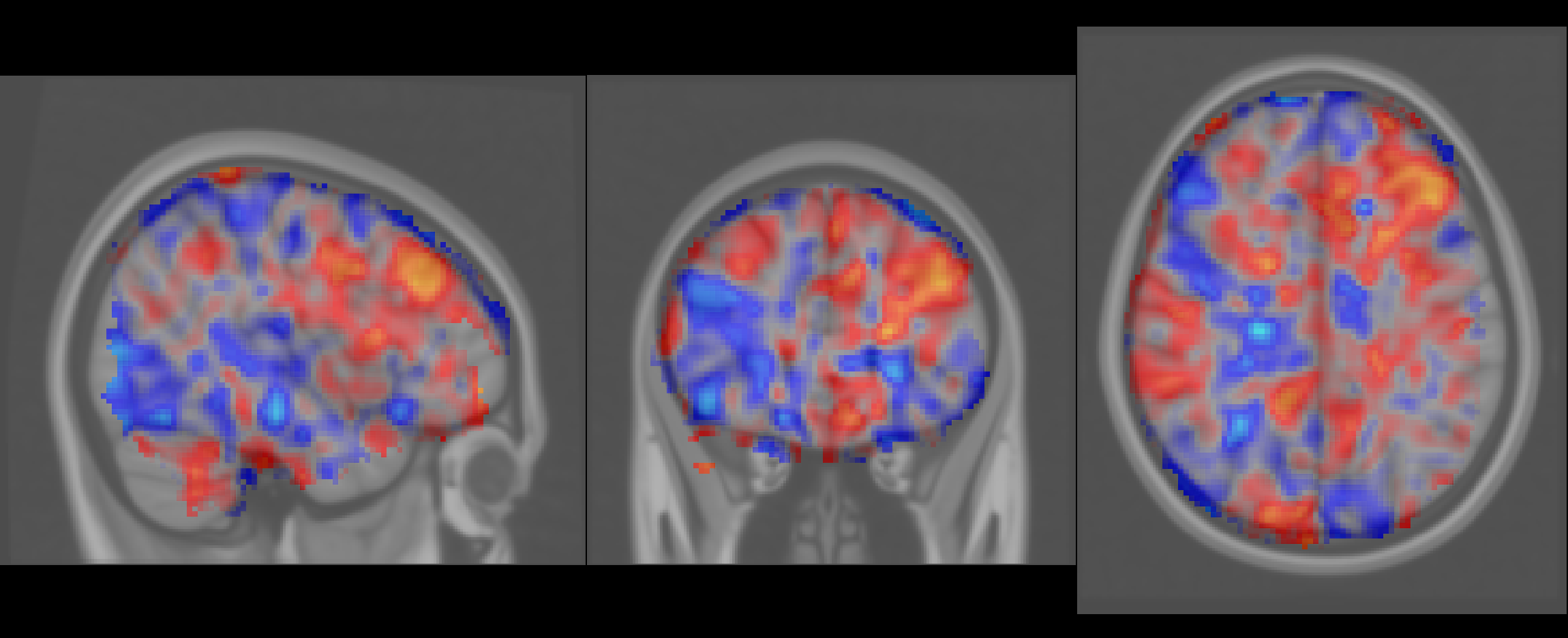

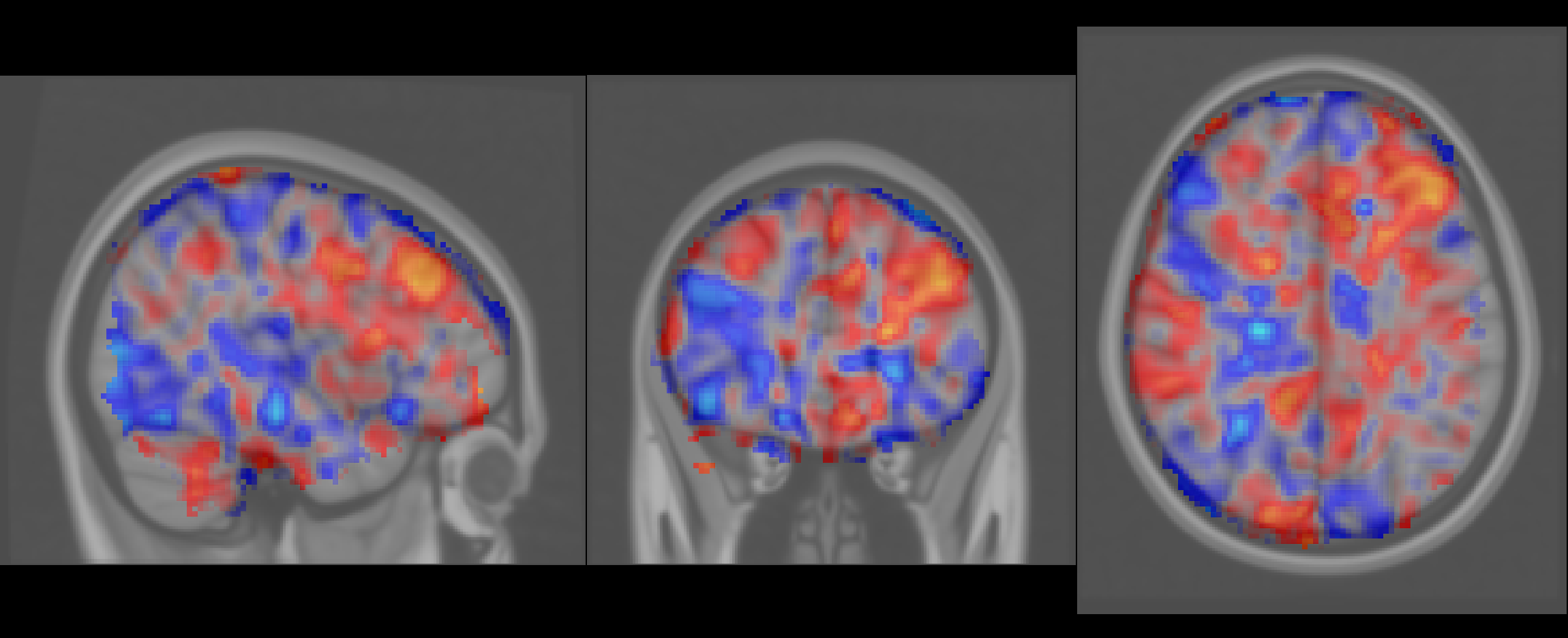


L

y = 28

z = 32

x = -40

ASD Glx/Cr

eFig 3.B

ASD GABA+/Cr

TD GABA+/Cr

TD > ASD GABA+/Cr

Z score

z = 10

z = 72

z = -42

4.5

-4.5

z = 46


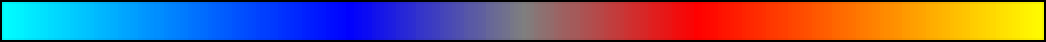

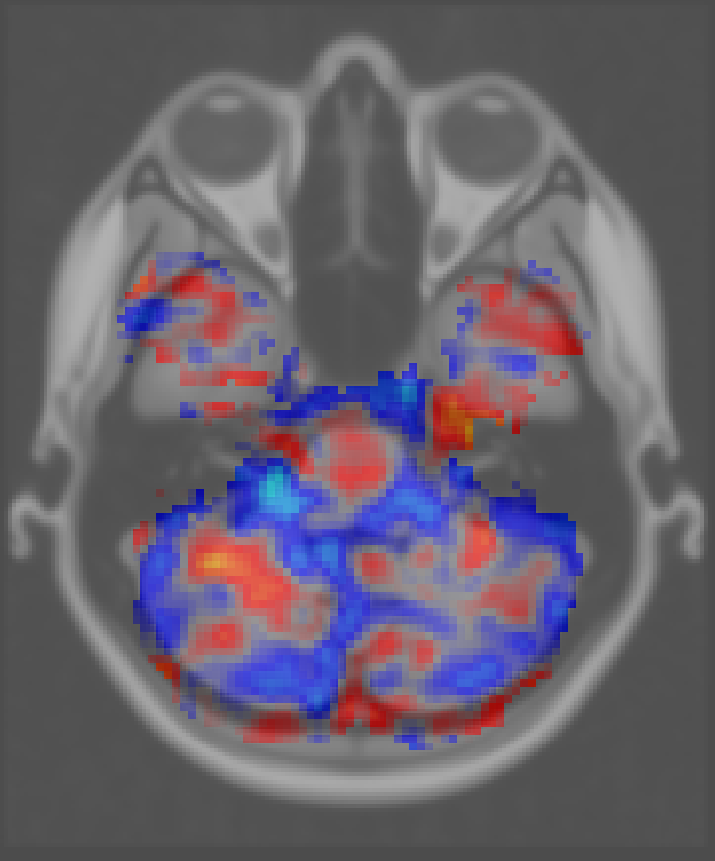


L


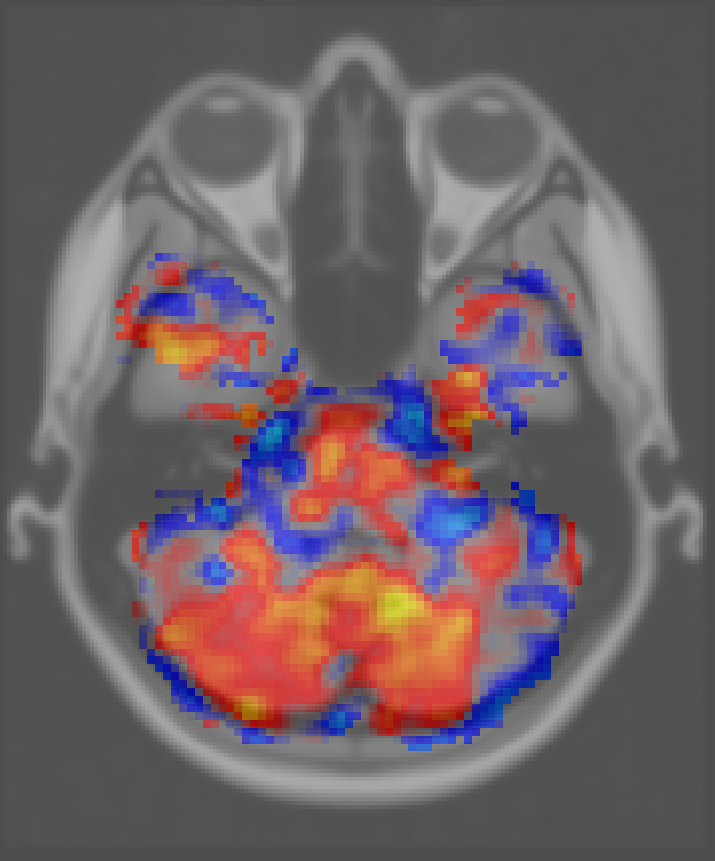


L


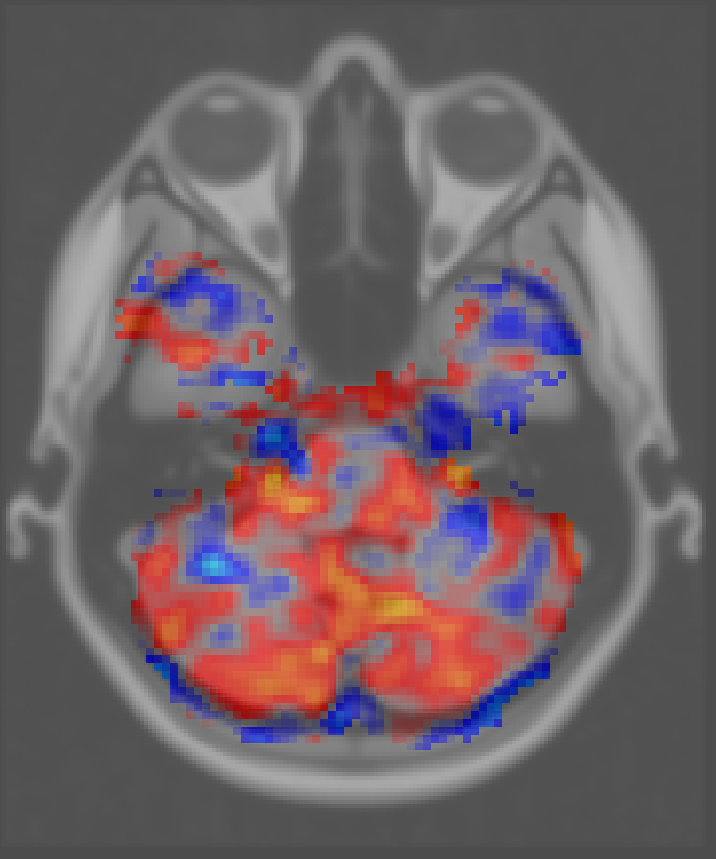


L


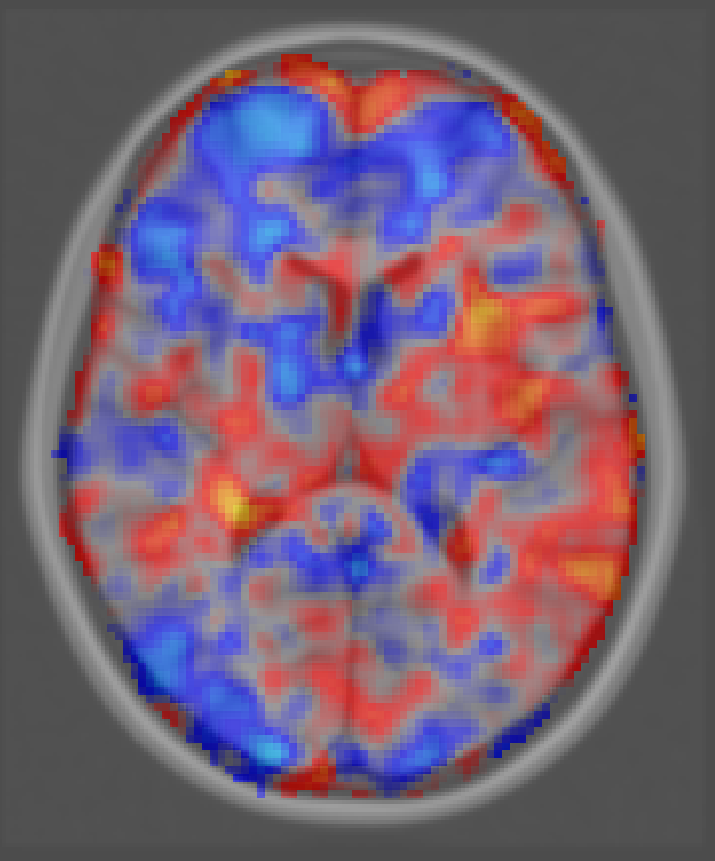

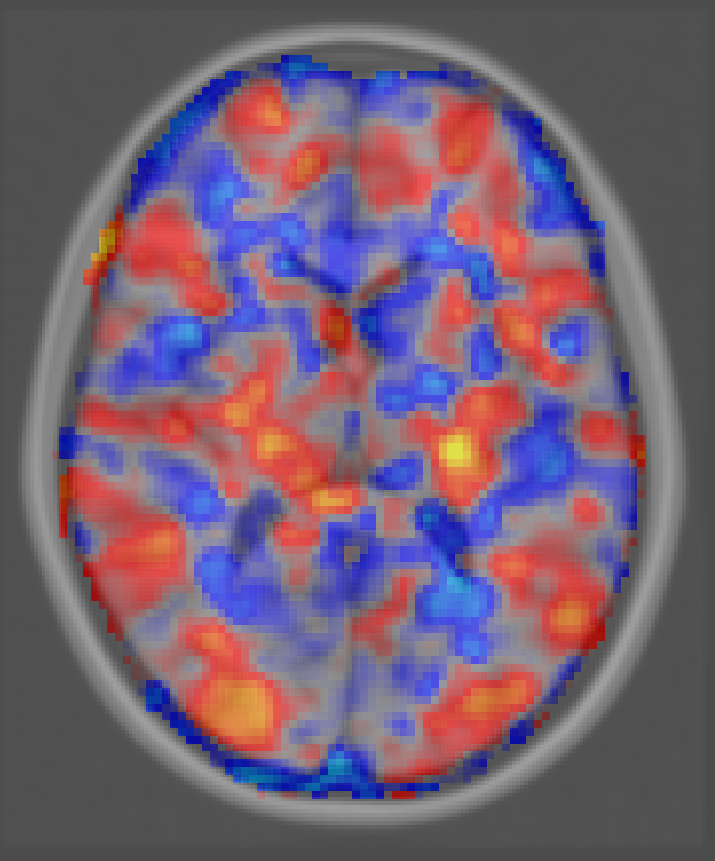

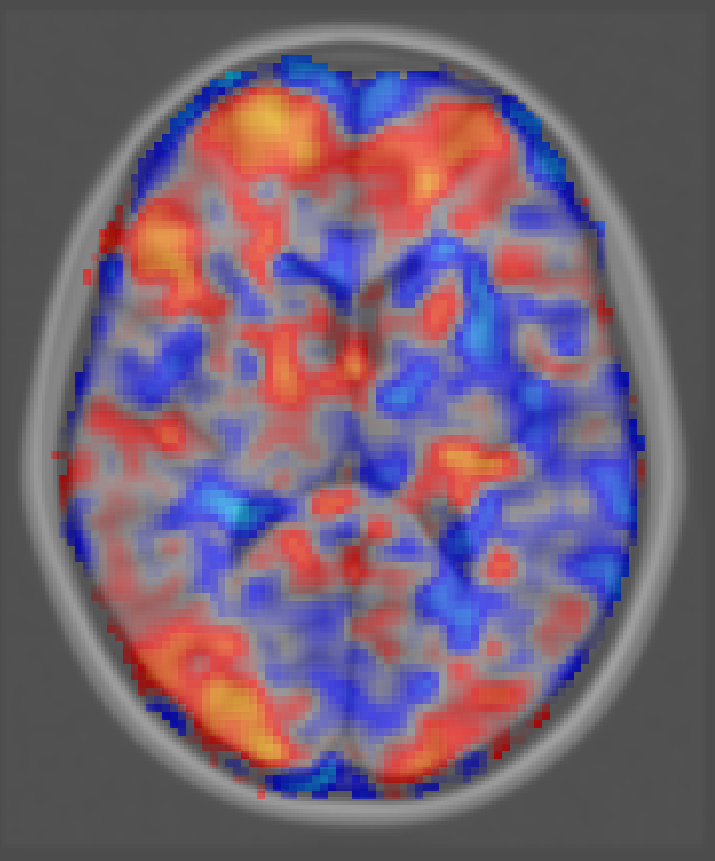

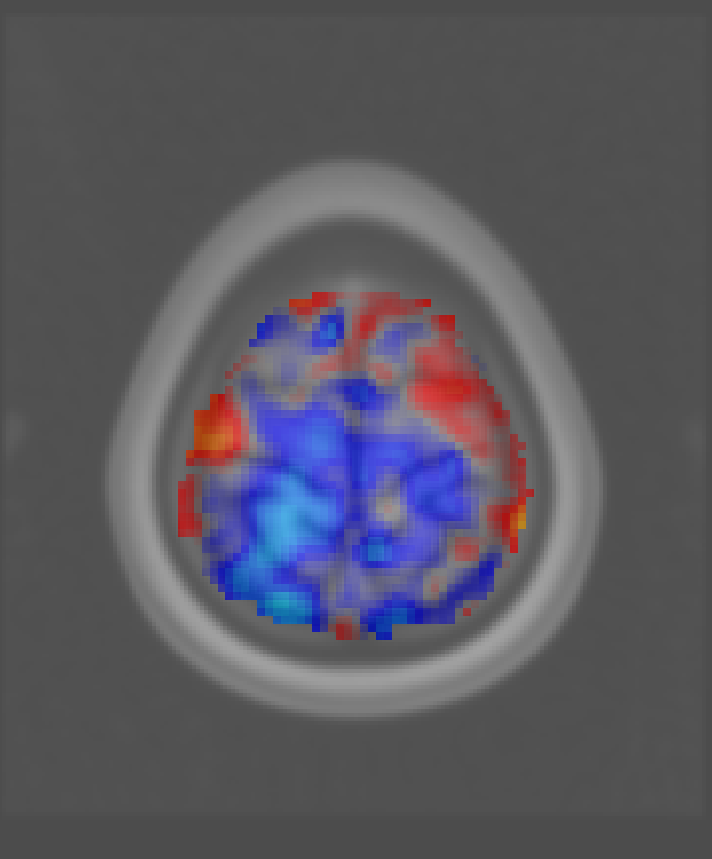

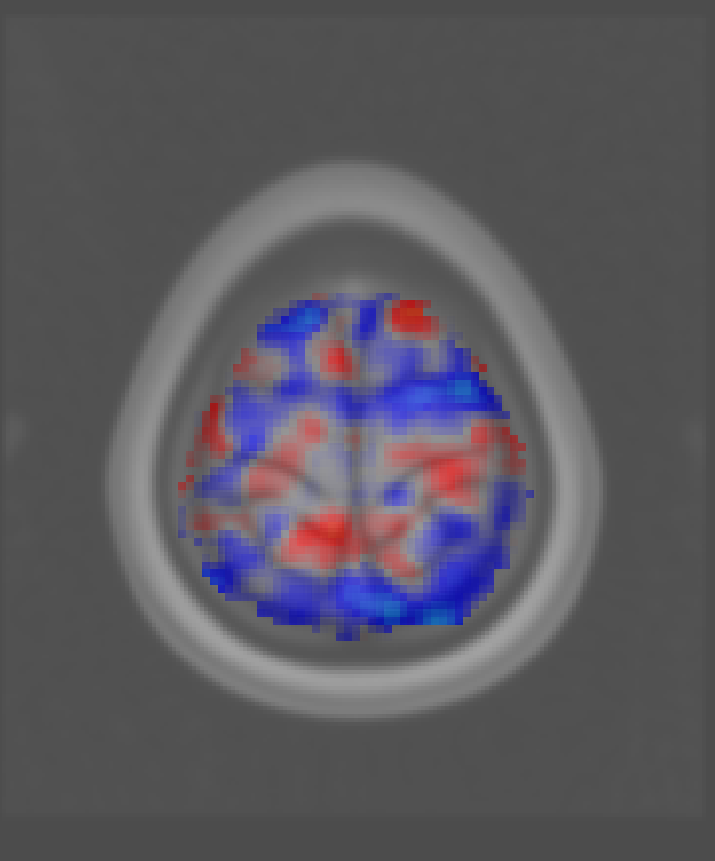

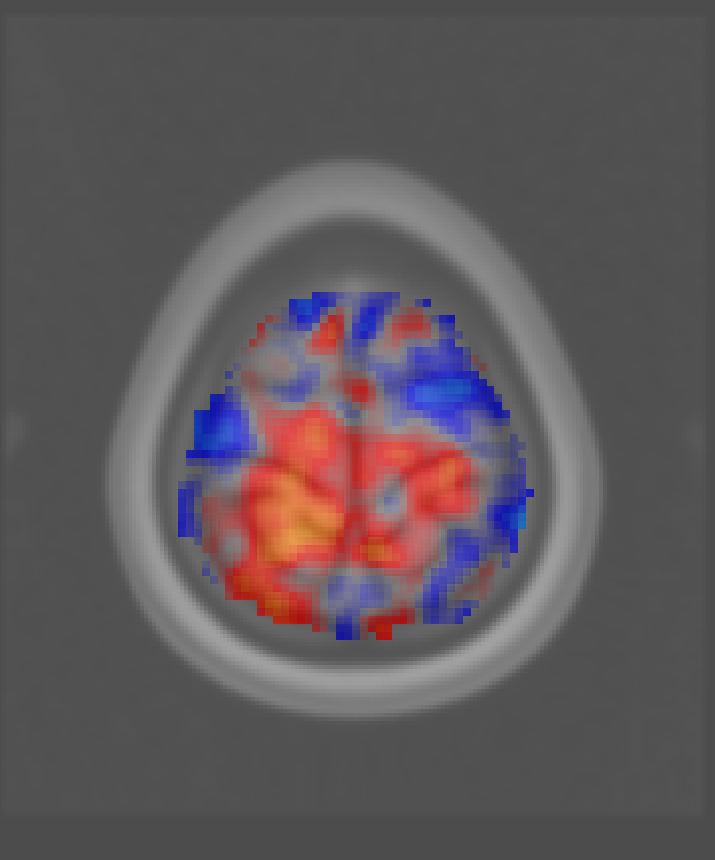

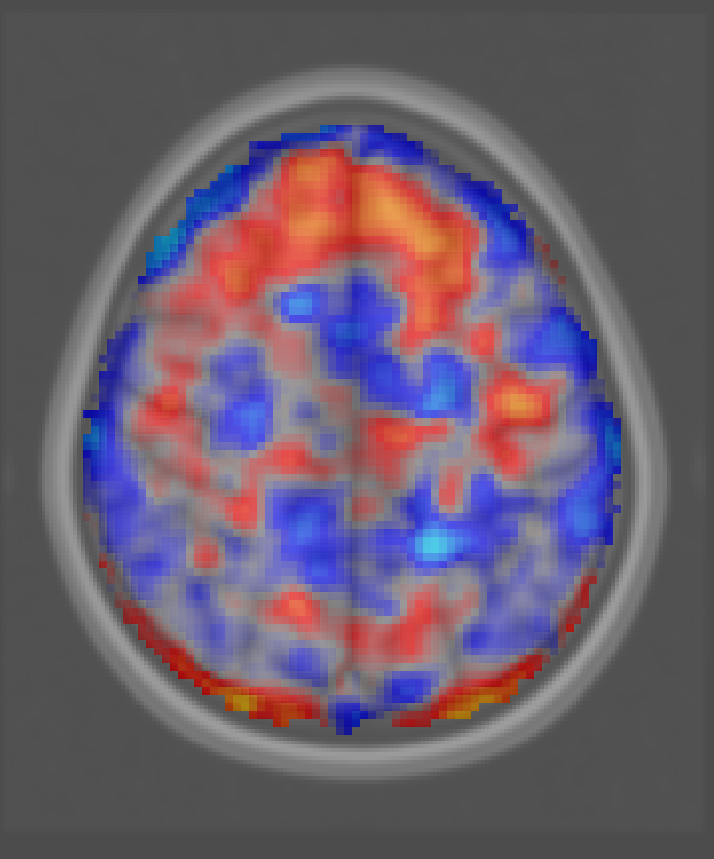

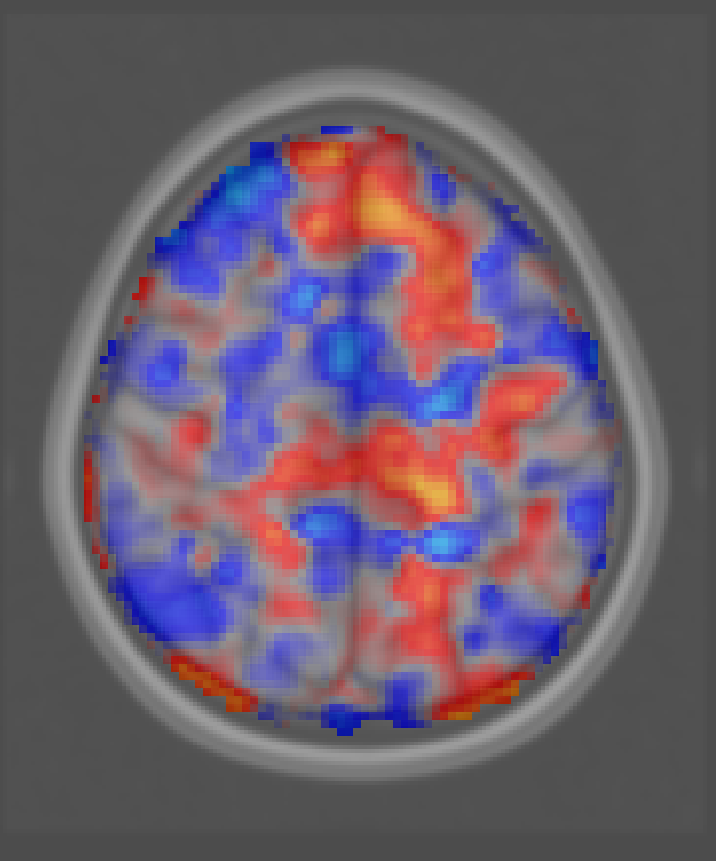

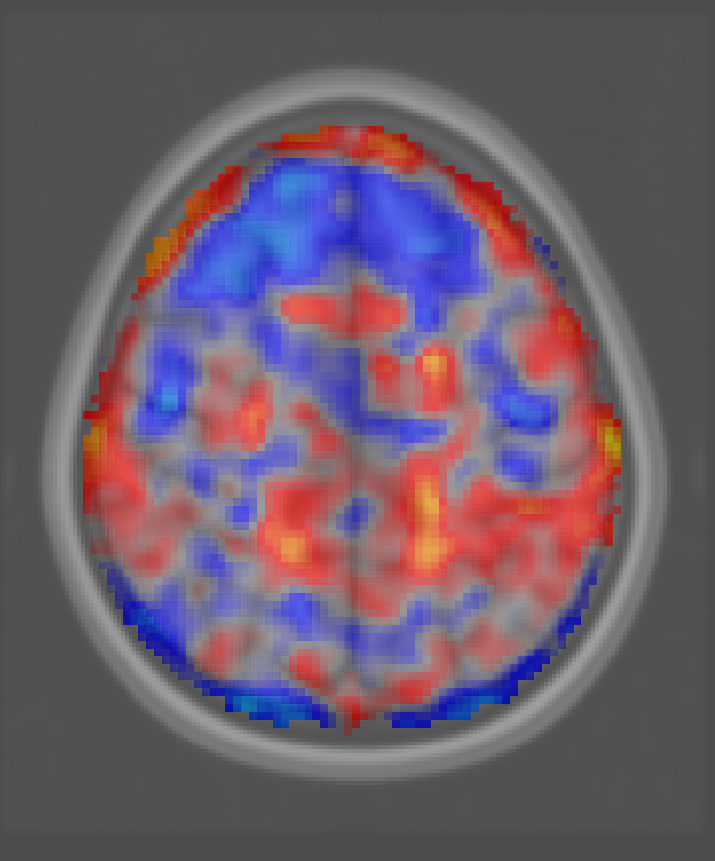


eFig 3.A

eFigure 4. Resting State Connectivity by Low/High Metabolite Level

Thalamic GABA+/Cr (A-G) and SSC Glx/Cr (H) were median split into low and high categories using all subjects. For the thalamus, significant between group differences in connectivity strength are only seen at the low GABA level (A, B, C, & E). For somatosensory Glx, between group connectivity differences are only seen in the high Glx level (H).


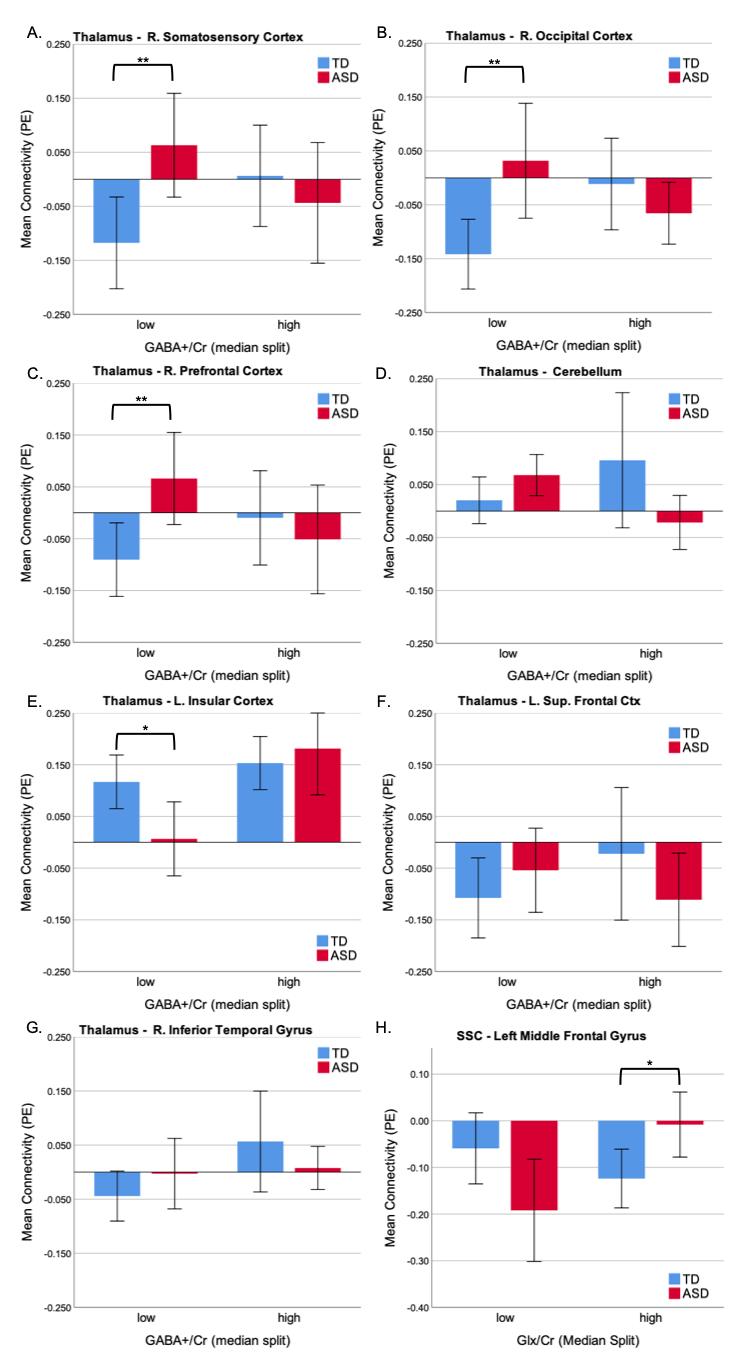


eFigure 5. Thalamocortical Connectivity Related to Sensory Over-Responsivity Severity

Correlation of functional connectivity between the thalamus and (A) left insular cortex (*r*=-0.41, *p*<0.05) and (B) right PFC (*r* =0.47, *p*<0.05, Fig. 3.B) controlling for anxiety.


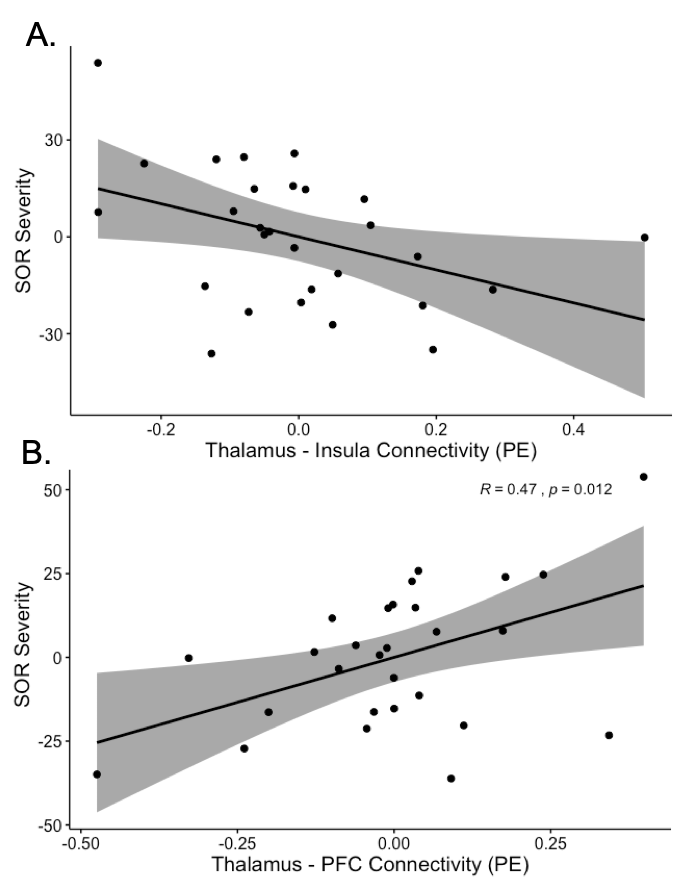

Supplement: Supplementary file 1 — Supplemental Material [file 41398_2020_1154_MOESM1_ESM.docx]
